# Chest pain without obstructive coronary artery disease: a case series

**DOI:** 10.1093/ehjcr/ytaa060

**Published:** 2020-04-24

**Authors:** Novalia P Sidik, Michael McDermott, Margaret B McEntegart, Colin Berry

**Affiliations:** y1 West of Scotland Heart and Lung Centre, Golden Jubilee National Hospital, Agamemnon Street, Glasgow G81 4DY, UK; y2 British Heart Foundation Glasgow Cardiovascular Research Centre, Institute of Cardiovascular and Medical Sciences, University of Glasgow, Glasgow, UK

**Keywords:** Coronary microvascular dysfunction, Ischaemia with no obstructive coronary artery disease, Microvascular angina, Vasospastic angina, Case series

## Abstract

**Background:**

Ischaemic heart disease is a leading cause of mortality in women. Even in those without obstructive coronary artery disease (CAD), women with angina continue to have increased mortality. There are gender differences in prevalence of different pathophysiologies, including functional disorders such as microvascular and vasospastic angina.

**Case summary:**

We describe four cases of angina in women with no obstructive CAD, in whom coronary function testing was performed. These four patients were diagnosed with disorders of coronary vasomotion, including vasospastic angina and different endotypes of microvascular angina.

**Discussion:**

This case series highlights the different mechanisms of ischaemia in the absence of obstructive CAD. Patients with angina and no obstructive CAD classified by computed tomography coronary angiography may have myocardial ischaemia due to microvascular angina, vasospastic angina, or both. Conventional investigations risk under-diagnosing, and as a consequence under-treating, patients with these conditions. Coronary function testing, in the form of diagnostic guidewire-based tests and adjunctive acetylcholine provocation, has proven to be critical in the accurate diagnoses and appropriate management of these patients.

**Learning points**

- Coronary vascular dysfunction (including microvascular angina and vasospastic angina) may cause ischaemia with no obstructive coronary artery disease, especially in women.
- An anatomical imaging mode of investigation risks overlooking coronary vascular dysfunction in patients presenting with angina.

## Introduction

Ischaemic heart disease (IHD) is a leading cause of premature morbidity and mortality.^1^^,^^2^ Although mortality from IHD has fallen in men, this is less apparent in younger women.^3^ Sex differences in the prevalence of distinct pathophysiologies of IHD may be relevant. Recent IHD guidelines reflect the diverse spectrum and aetiopathogenesis of patients with chronic coronary syndromes,^4^ with new emphasis on functional disorders such as microvascular angina and vasospastic angina. These are more common in women.^5^ The WISE study, in particular, showed that one-third of cardiac deaths in their cohort occurred in women without obstructive coronary artery disease (CAD).^6^ Ischaemia with no obstructive CAD is increasingly recognized as an important entity, and the underlying mechanisms merit further study.

This report describes four female patients with angina in whom obstructive CAD was ruled out by computed tomography coronary angiography (CTCA). Each patient gave informed consent to enrol in a randomized controlled trial (CorCTCA, ClinicalTrials.gov NCT03477890) assessing the effect of a clinical strategy of stratified medicine on diagnosis, treatment, and well-being. They were invited to undergo invasive coronary angiography (CAG) and adjunctive tests of coronary vascular function as part of the study protocol. The latter involved the measurement of coronary flow reserve (CFR) and index of microvascular resistance (IMR) using a coronary thermodilution technique with a pressure- and temperature-sensitive diagnostic coronary guidewire,^7^ and acetylcholine provocation testing with infusions of incremental acetylcholine concentrations (10^−6 ^M, 10^−5 ^M, 10^−4 ^M), each delivered over 2 min. The thermodilution technique requires 3 mL of saline at room temperature to be injected briskly into the coronary artery to obtain a resting thermodilution curve. Three resting thermodilution curves are obtained to derive a mean transit time. This is repeated during hyperaemia, which is induced by adenosine, to obtain three hyperaemic thermodilution curves. Coronary flow reserve and IMR can then be calculated. This stratified medicine protocol^5^ is supported by contemporary practice guidelines.^4^

## Timeline

**Table:** 

	Patient 1	Patient 2	Patient 3	Patient 4
Time 0—computed tomography coronary angiography (CTCA)				
Duration of symptoms	6 years	2 years	2 years	9 months
CTCA findings	Normal coronary arteries	Normal coronary arteries	Non-obstructive coronary artery disease (CAD)	Plaque in left anterior descending artery (LAD)
Time 1—invasive coronary angiography (CAG) and coronary function testing				
CAG findings	Normal coronary arteries	Myocardial bridging in mid-LAD	Non-obstructive CAD	Tortuous coronary arteries
Coronary function test	Coronary flow reserve (CFR) 5.0	CFR 1.7	CFR 1.5	CFR 1.6
	Index of microvascular resistance (IMR) 12	IMR 24	IMR 11	IMR 41
Acetylcholine provocation test	Epicardial vasospasm with reproduction of chest pain	Microvascular spasm with reproduction of chest pain and electrocardiogram (ECG) changes	Reproduction of chest pain with ECG changes	No response
Randomization group	Standard, angiography-guided arm	Standard, angiography-guided arm	Standard, angiography-guided arm	Standard, angiography-guided arm
Management	Likely non-cardiac chest pain; no changes to medication	Probable non-cardiac chest pain; no changes to medication	Possible microvascular angina; statin therapy started	Possible microvascular angina; no changes to medication
Time 2—follow-up				
Time to follow-up (months)	12	12	12	6
Diagnosis	Vasospastic angina	Microvascular angina	Microvascular angina	Microvascular angina
Management	Tildiem 200 mg	Verapamil 240 mg and statin therapy	Amlodipine and bisoprolol switched to verapamil 120 mg and ramipril 2.5 mg	—

## Case presentation

### Patient 1

A 46-year-old woman presented to the cardiology clinic with a 6-year history of chest tightness radiating to her left shoulder, occurring predominantly on exertion but also at rest. She reported difficulty climbing stairs due to her symptoms. She had not been prescribed any cardiac medication and her medical therapy consisted only of duloxetine for depression and pregabalin for chronic back pain. Her past medical history included emphysema, depression, osteoarthritis, irritable bowel syndrome with previous normal gastroscopy and sigmoidoscopy, and previous cholecystectomy. She underwent a treadmill exercise tolerance testing (ETT) but was only able to exercise for 3 min and 3 s, stopping due to leg fatigue. She had no chest pain or changes on her electrocardiogram (ECG). The ETT was deemed inconclusive and she subsequently had a CTCA which showed normal coronary arteries. The diagnosis made by her attending cardiologist was non-cardiac chest pain.

**Figure 1:**
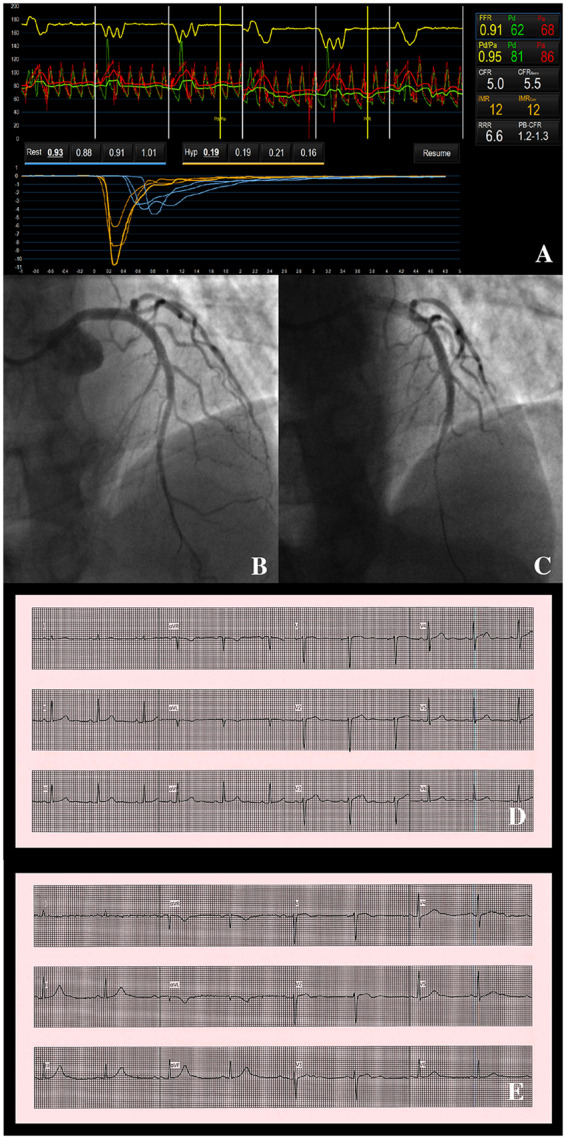
Patient 1—coronary function test results, angiographic changes on acetylcholine provocation testing and electrocardiographic findings. Coronary vascular function testing was performed in the LAD using the thermodilution technique with the Coroventis^®^ software. Coronary flow reserve and index of microvascular resistance were both normal (*A*). The LAD was unobstructed (*B*), but on acetylcholine provocation testing, there was epicardial vasospasm from the mid to distal segment (*C*). The patient’s electrocardiogram (*E*) was largely unchanged from baseline (*D*).

Invasive CAG confirmed the CTCA findings of angiographically normal coronary arteries. Coronary vascular function testing was performed in the left anterior descending coronary artery (LAD) and the IMR (12, normal < 25) and CFR (5.0, normal > 2.0) were normal. On acetylcholine provocation testing, epicardial vasospasm was observed, accompanied by transient chest pain, but without ECG changes (*Figure 1*). The vasospasm and chest pain resolved with administration of intracoronary glyceryl trinitrate (GTN). These findings were consistent with a diagnosis of vasospastic angina.

The patient had been randomized to the standard, angiography-guided arm of the trial, and therefore the cardiologist was unaware of the coronary function findings. A diagnosis of non-cardiac chest pain was made.

At 12 months, she reported persistent chest symptoms. Her sublingual GTN spray, which had been discontinued by her primary care physician, was restarted and she was commenced on diltiazem.

### Patient 2

A 42-year-old woman presented with a 2-year history of typical angina following the onset of premature menopause. At the time of presentation, she described angina consistent with Canadian Cardiovascular Society (CCS) Class II severity, with predictable chest tightness when walking up hills. She reported no symptoms at rest. The patient had been prescribed propranolol 10 mg three times daily for anxiety and an oestrogen patch for hormone replacement therapy. She was an ex-smoker and her past medical history included obesity, migraine, and depression. She underwent an ETT, exercising for 6 min and 23 s with reproduction of chest pain at 4 min but no ECG changes. The test was stopped due to dyspnoea and fatigue. Her attending cardiologist felt that CAD could not be excluded and referred her for a CTCA, which showed normal coronary arteries.

**Figure 2:**
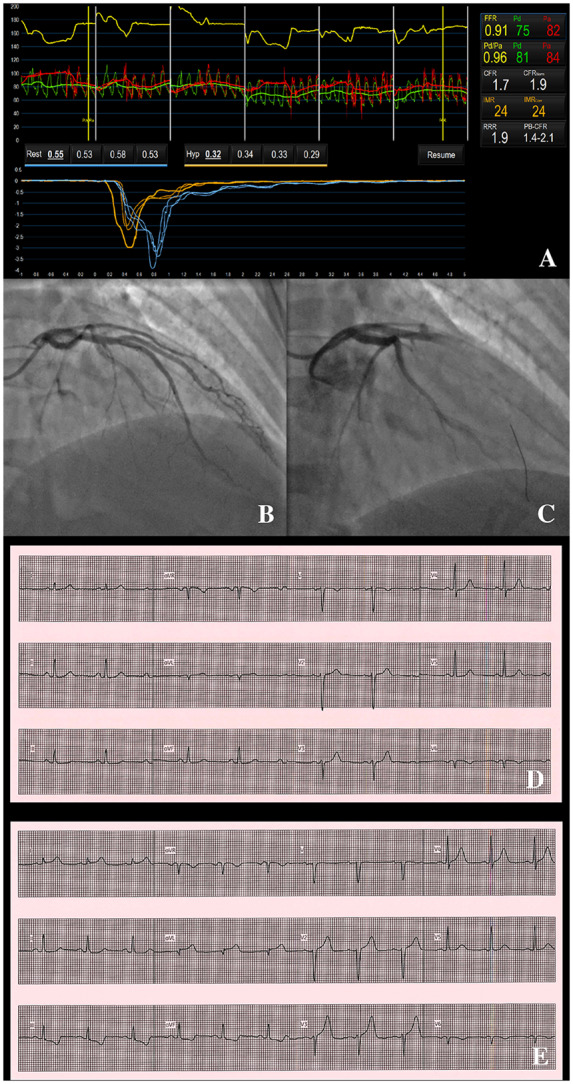
Patient 2—coronary function test results, angiographic changes on acetylcholine provocation testing and electrocardiographic changes. Coronary vascular function testing was performed in the LAD using the thermodilution technique with the Coroventis^®^ software. Coronary flow reserve was low at 1.7 (*A*). The LAD was unobstructed (*B*). On acetylcholine provocation testing, there was a loss of flow in the LAD (*C*), accompanied by high lateral ST elevation and inferior ST depression (*E*), which were not present at baseline (*D*).

Invasive CAG revealed myocardial bridging in the mid-LAD. Coronary flow reserve on coronary function testing was low at 1.7, with a borderline IMR of 24. On acetylcholine provocation testing, there was a transient reduction in antegrade flow in the LAD without evidence of epicardial vasospasm (*Figure 2*). The patient experienced transient chest pain and the ECG disclosed ST elevation in leads I and aVL with reciprocal ST depression in leads II, III, and aVF. The angiographic appearance following acetylcholine, accompanied by the reproduction of her usual symptoms and ECG changes, was consistent with microvascular spasm, an endotype of microvascular angina.

The patient had been randomized to the standard, angiography-guided arm of the trial. She was diagnosed with probable non-cardiac chest pain. No changes to her medication were made.

At 12 months, the patient was unblinded. Her angina status remained consistent with CCS Class II severity and she was prescribed verapamil 240 mg daily and statin therapy. Her primary care physician had referred her to the cardiac rehabilitation service for supervised exercise. Four months later, she reported an improvement in her angina. Her exercise tolerance had improved and she only had symptoms when the weather was particularly cold. Her Seattle Angina Questionnaire—Physical Limitation (SAQ-PL) score had improved from 55 to 72 following the introduction of verapamil.

### Patient 3

A 59-year-old woman presented with a 2-year history of left-sided chest tightness radiating down her left arm, occurring predominantly on exertion but also at rest. She reported symptoms on walking up an incline. She was treated with amlodipine 5 mg daily for hypertension. She also had a history of hyperlipidaemia, previous hysterectomy, fibromyalgia, and sciatica. Following the onset of stable chest pain, her primary care physician had prescribed bisoprolol 2.5 mg daily, which had slightly improved her symptoms. The CTCA scan disclosed minor plaque in the right coronary artery (RCA) and a calcified, non-obstructive (<50%) stenosis in the proximal LAD.

**Figure 3:**
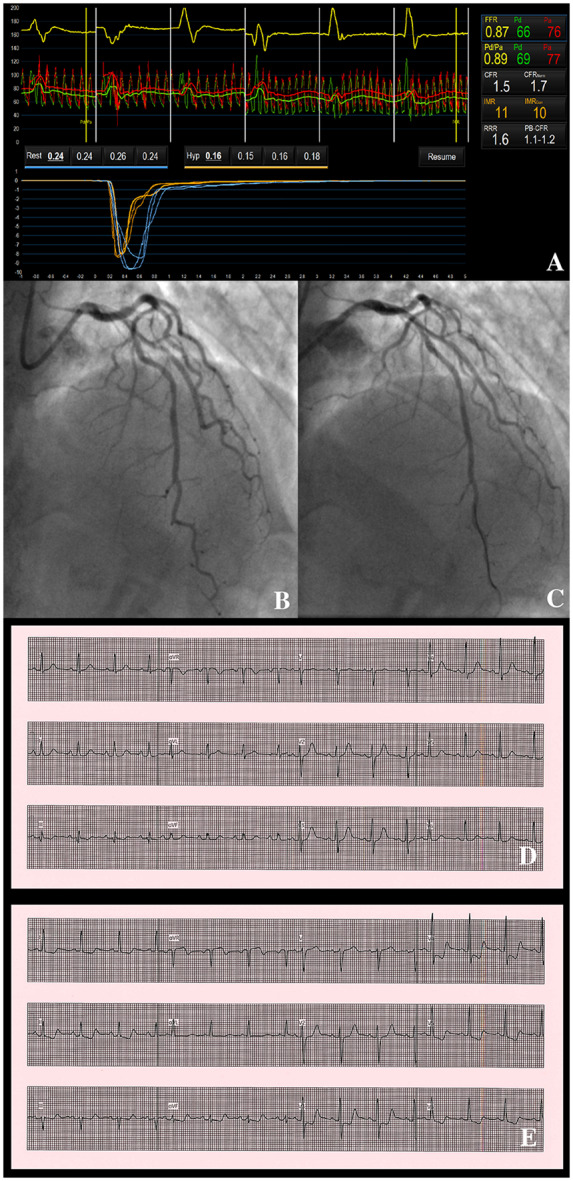
Patient 3—coronary function test results, angiographic findings on acetylcholine provocation testing and electrocardiographic changes. Coronary vascular function testing was performed in the LAD using the thermodilution technique with the Coroventis^®^ software. Coronary flow reserve was low at 1.5 (*A*). There was mild, non-obstructive disease in the LAD (*B*). There was no change in flow or vessel diameter on acetylcholine challenge (*C*), however, the patient had chest pain with anterior ST depression (*E*), which was not present at baseline (*D*).

Invasive CAG confirmed non-obstructive plaque in the RCA and proximal to mid-LAD. Fractional flow reserve in the LAD was 0.87 (normal ≥ 0.80), confirming non-obstructive CAD. On coronary function testing, CFR was 1.5 and IMR was 11. Acetylcholine provocation disclosed normal angiographic responses, but the patient experienced chest pain with anterior ST depression (*Figure 3*). This resolved with intracoronary GTN. There was evidence of both reduced coronary vasorelaxation (reduced CFR) and microvascular spasm (chest pain with ECG changes, despite the absence of change in coronary flow on angiography), consistent with a diagnosis of microvascular angina.

The patient had been randomized to the standard, angiography-guided arm of the trial. Her attending cardiologist felt that microvascular angina was possible, but did not make any changes to her antianginal medication. Statin therapy was prescribed.

At 12 months, the patient was unblinded. She reported persistent angina (CCS Class III). She was given a diagnosis of microvascular angina and her amlodipine and bisoprolol were changed to verapamil 120 mg daily and ramipril 2.5 mg daily. Three months later, she reported an improvement in her symptoms, although she continued to have CCS Class II angina. Her SAQ-PL score had improved slightly from 44 to 47.

### Patient 4

A 51-year-old woman presented with a 9-month history of atypical chest pain. She reported predominantly exertional, but also non-exertional left-sided chest pain radiating to her jaw. Her symptoms would occur on walking up an incline. She had not been prescribed any cardiac medication. She underwent an ETT and developed chest pain at 1 min and 23 s which worsened with continued exercise. The test was stopped at 6 min and 22 s due to limiting chest pain. There were no ECG changes. A subsequent CTCA scan revealed plaque in the proximal LAD.

**Figure 4:**
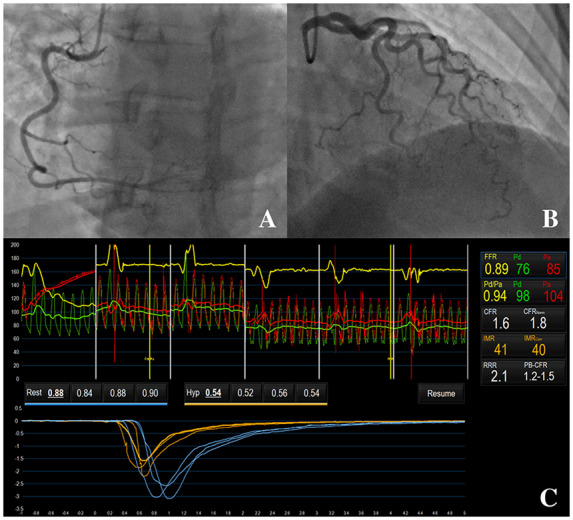
Patient 4—coronary angiogram and coronary function test results. Coronary angiography revealed tortuous coronary arteries that were unobstructed (*A* and *B*). Coronary vascular function testing was performed in the LAD using the thermodilution technique with the Coroventis^®^ software. Coronary flow reserve was low at 1.6 and index of microvascular resistance was high at 41 (*C*).

Invasive CAG revealed tortuous coronary arteries with no obstructive disease. On coronary function testing, CFR was 1.6 and IMR was 41 (*Figure 4*). These findings reflected reduced coronary vasorelaxation and increased microvascular resistance, which were consistent with a diagnosis of microvascular angina. Coronary reactivity testing with acetylcholine was negative.

The patient was randomized to the standard, angiography-guided arm of the trial. The cardiologist was blinded to the results of the coronary vascular function test, and the decision to prescribe antianginal therapy was left to her primary care physician.

At 6 months, the patient reported worsening anginal symptoms and lethargy. She had not been prescribed any antianginal therapy. Blinded follow-up is ongoing.

### Discussion

A clinical strategy of anatomical imaging of the coronary arteries, which is currently recommended as the first-line test in UK practice guidelines,^8^^,^^9^ does not take into account the disorders of coronary vasomotion and related susceptibility to myocardial ischaemia. Patients with angina and no obstructive CAD classified by CTCA may have myocardial ischaemia due to microvascular angina, vasospastic angina, or both. Conventional investigations risk under-diagnosing, and as a consequence under-treating, patients with these conditions. Quality of life, anginal symptoms, and prognosis may be persistently impaired when the management of these patients is guided solely by an anatomical strategy.^5^

In contrast, the practice guidelines of the European Society of Cardiology now recommend assessing coronary vascular function in patients with angina and no obstructive CAD.^4^ Stratified medicine promotes patient-centred care and is prognostically informative.^10^ Distinguishing the different endotypes of coronary vascular dysfunction (e.g. microvascular spasm vs. high microvascular resistance) leads to personalized therapy, which in turn leads to improved symptoms.^5^ First-line therapy for patients with microvascular angina consists of a beta-blocker, unless they have predominantly microvascular spasm, which should be treated like vasospastic angina with a calcium channel blocker. Add-on therapy includes calcium channel blockers and nicorandil in microvascular angina and a long-acting nitrate in vasospastic angina.

This case series highlights the different mechanisms of ischaemia in the absence of obstructive CAD. Coronary function testing, in the form of diagnostic guidewire-based tests and adjunctive acetylcholine provocation, has proven to be critical in the accurate diagnoses and appropriate management of these patients. The CorCTCA study will determine the prevalence of these conditions in a comparatively unselected patient population and whether stratified medicine will benefit these patients.

## Lead author biography

**Figure:**
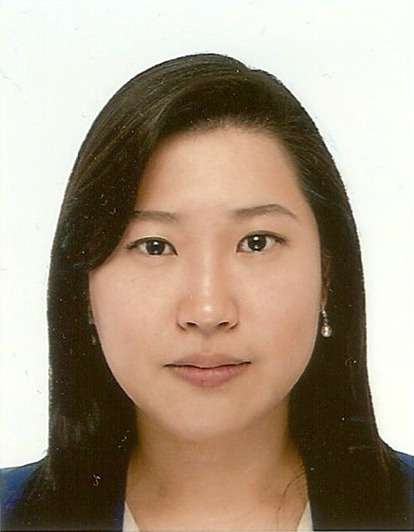


Dr Novalia P. Sidik is a British Heart Foundation Clinical Research Fellow and a cardiology specialist registrar in the West of Scotland. She is based at the University of Glasgow and the National Heart and Lung Centre at Golden Jubilee National Hospital in Glasgow, Scotland. Her research focuses on coronary physiology and coronary intervention.

## Supplementary material

Supplementary material is available at *European Heart Journal - Case Reports* online.

## Funding

N.P.S. is a British Heart Foundation (BHF) Clinical Research Training Fellow (FS/17/26/32744). Her research is also supported by a BHF Centre of Research Excellence award (RE/18/6134217) and a subvention grant from the Chief Scientist Office of the Scottish Government under the terms of section 47 of the National Health Service (Scotland) Act 1978. C.B. has research support from the British Heart Foundation (PG/17/2532884, FS/17/26/32744, RE/18/6134217) and Medical Research Council (MR/S005714/1).

**Slide sets:** A fully edited slide set detailing this case and suitable for local presentation is available online as Supplementary data.

**Consent:** The author/s confirm that written consent for submission and publication of this case report including image(s) and associated text has been obtained from the patient in line with COPE guidance.

**Conflict of interest:** C.B. is employed by the University of Glasgow which holds consultancy and/or research agreements with companies that have commercial interests in the diagnosis and treatment of ischaemic heart disease. The companies include Abbott Vascular, AstraZeneca, Boehringer Ingelheim, GSK, HeartFlow, Menarini Pharmaceuticals, Novartis, and Siemens Healthcare. None of these companies have had any involvement with this study. All other authors declared no conflict of interest.

## Supplementary Material

ytaa060_Supplementary_Slide-Set
