# Supplementary material for: Chest pain without obstructive coronary artery disease: a case series
Source: Eur Heart J Case Rep. 2020 Apr 24;4(3):1–6. doi: 10.1093/ehjcr/ytaa060 (PMC7319819; doi:10.1093/ehjcr/ytaa060)
Supplement: ytaa060_Supplementary_Slide-Set [file ytaa060_supplementary_slide-set.pptx]

## Slide 1
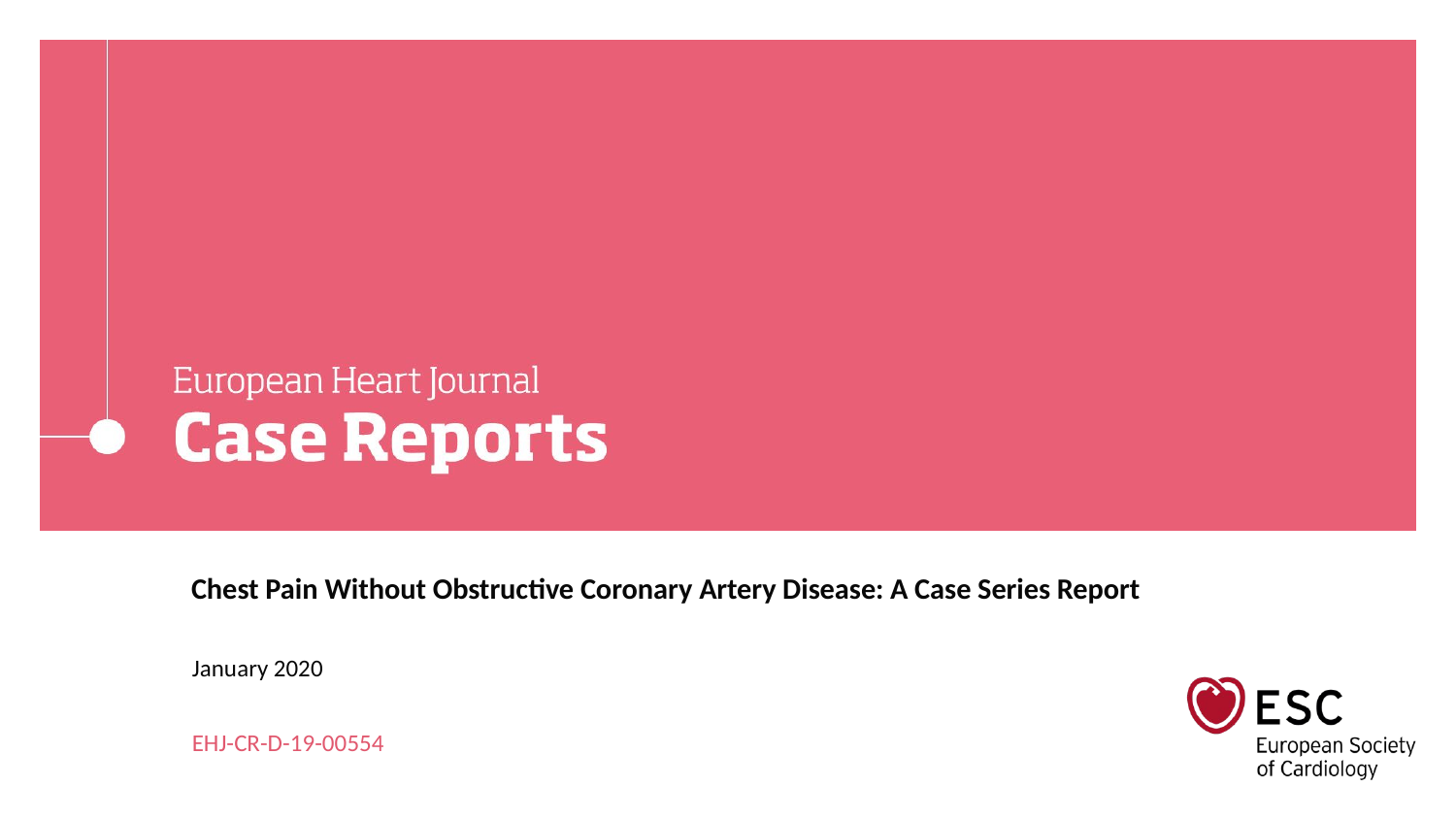

# Chest Pain Without Obstructive Coronary Artery Disease: A Case Series Report
January 2020
EHJ-CR-D-19-00554

## Slide 2
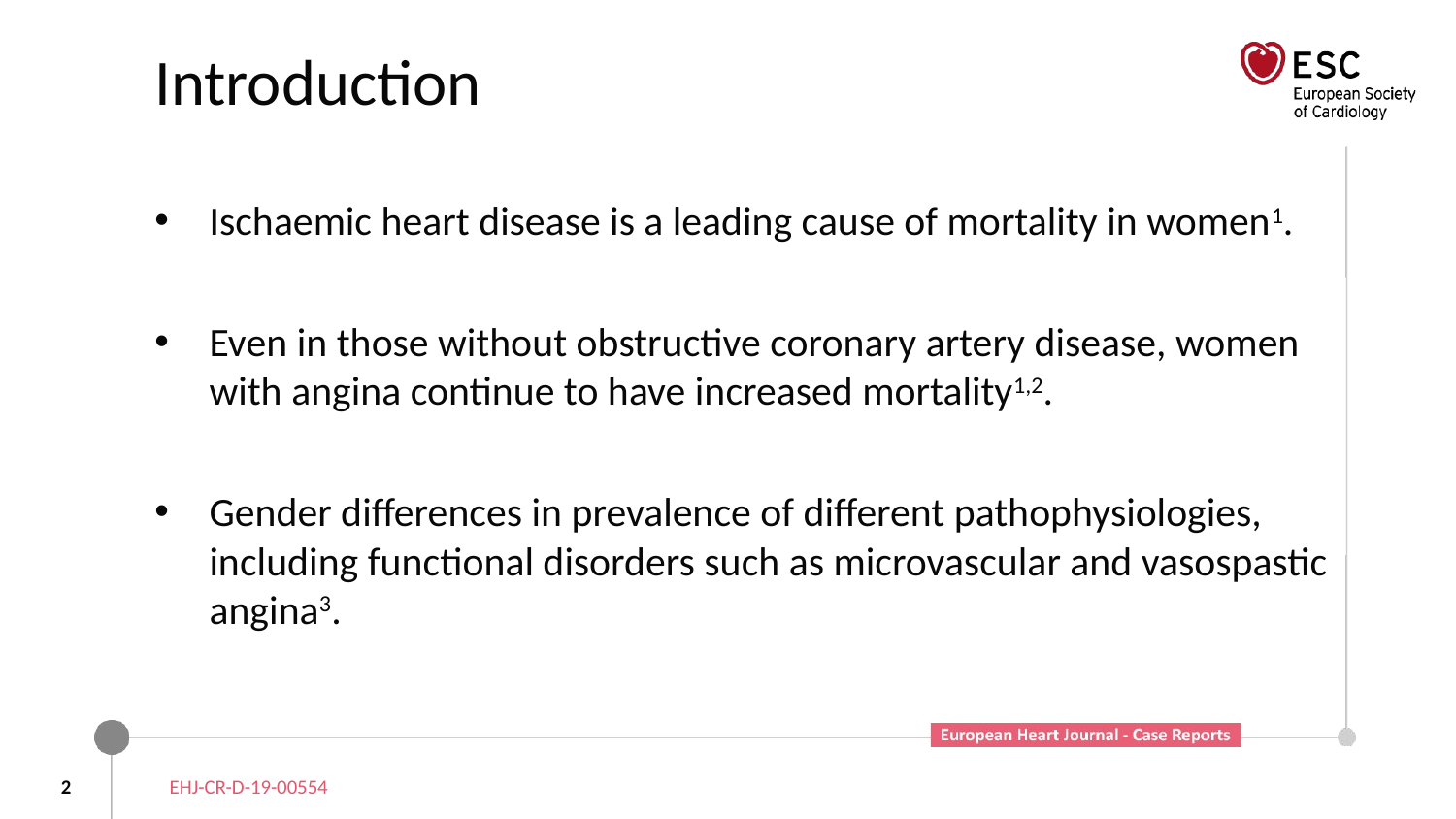

# Introduction
Ischaemic heart disease is a leading cause of mortality in women1.
Even in those without obstructive coronary artery disease, women with angina continue to have increased mortality1,2.
Gender differences in prevalence of different pathophysiologies, including functional disorders such as microvascular and vasospastic angina3.
2
EHJ-CR-D-19-00554

## Slide 3
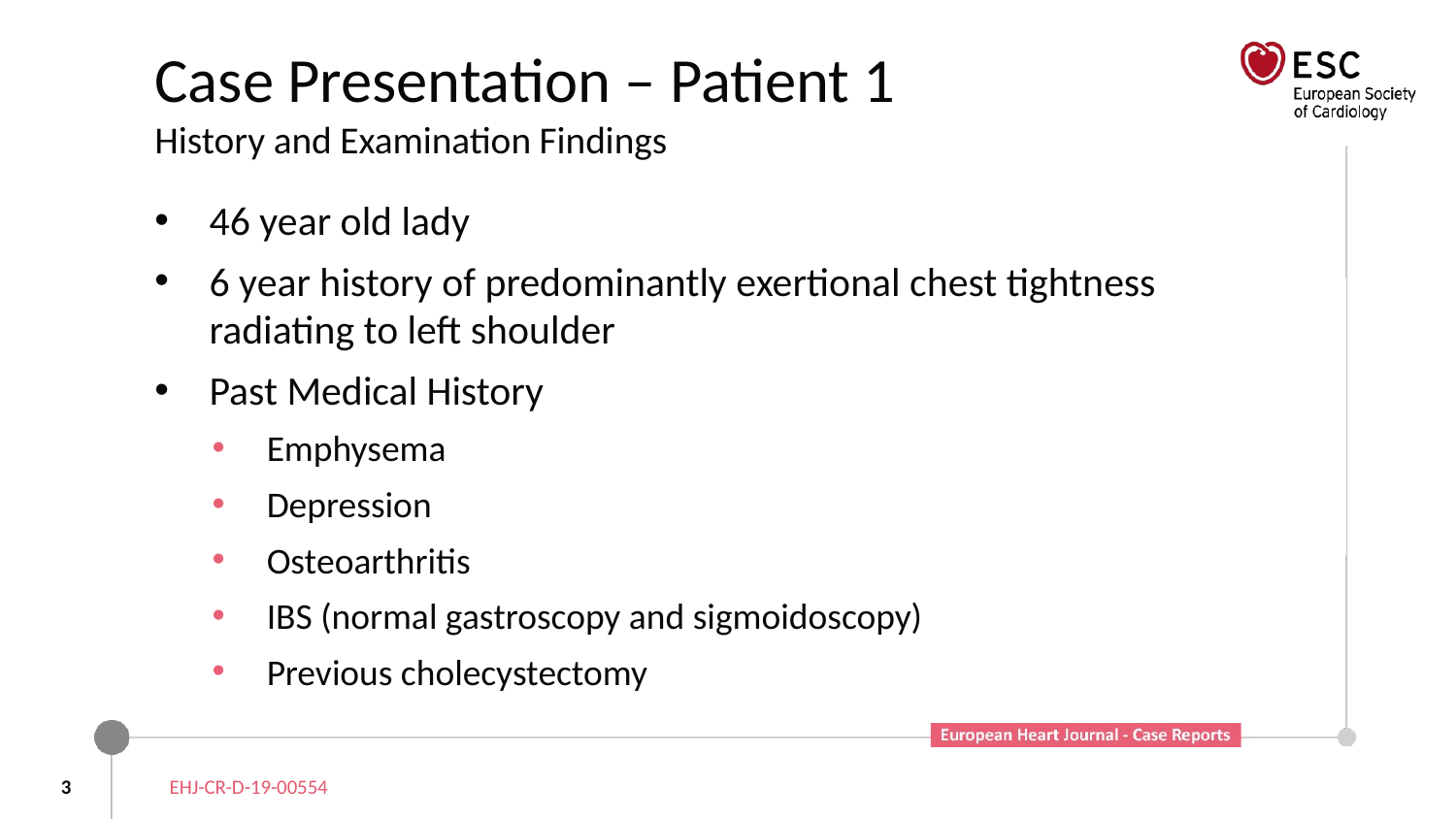

# Case Presentation – Patient 1 History and Examination Findings
46 year old lady
6 year history of predominantly exertional chest tightness radiating to left shoulder
Past Medical History
Emphysema
Depression
Osteoarthritis
IBS (normal gastroscopy and sigmoidoscopy)
Previous cholecystectomy
3
EHJ-CR-D-19-00554

## Slide 4
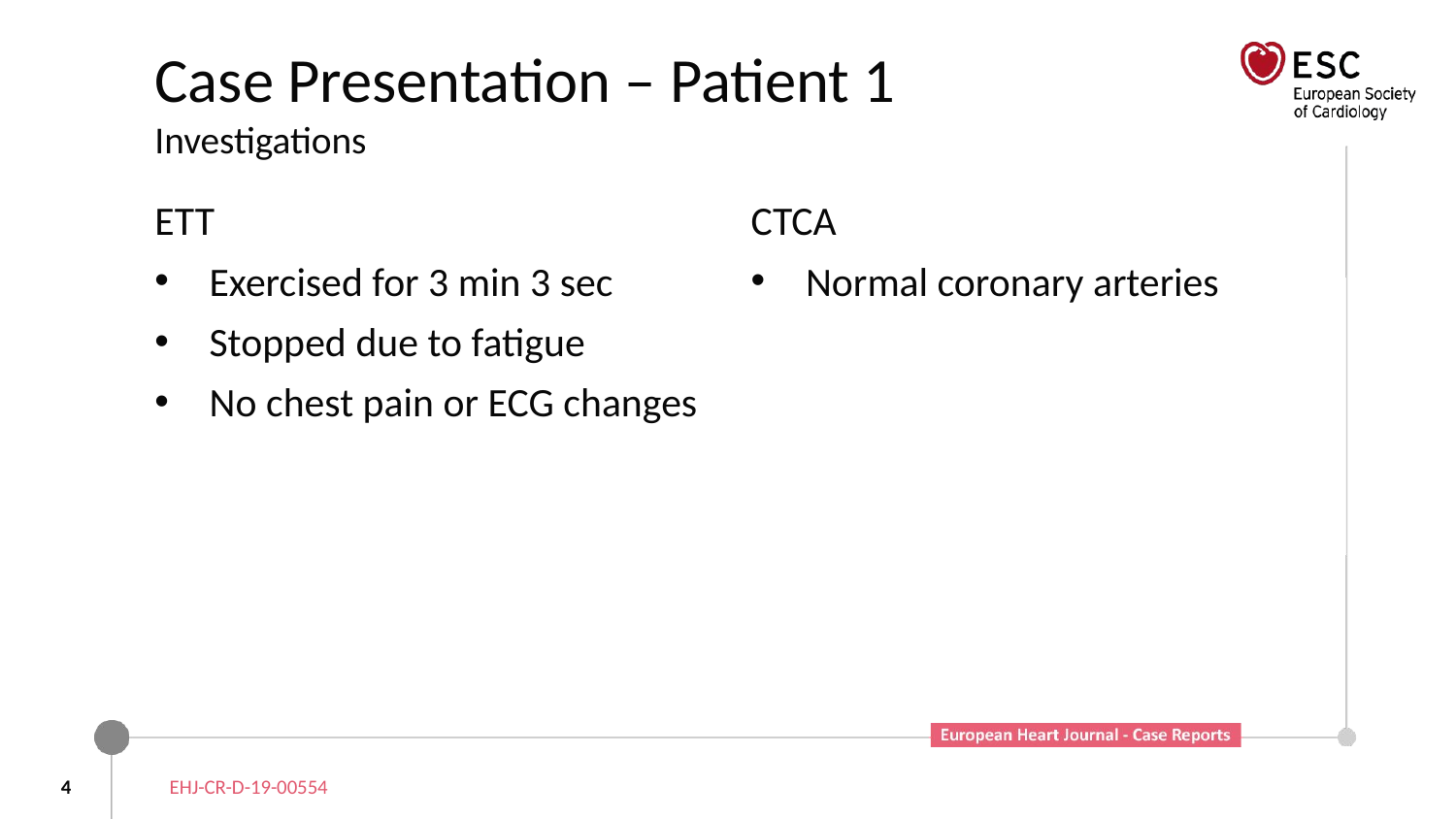

# Case Presentation – Patient 1 Investigations
ETT
Exercised for 3 min 3 sec
Stopped due to fatigue
No chest pain or ECG changes
CTCA
Normal coronary arteries
4
EHJ-CR-D-19-00554

## Slide 5
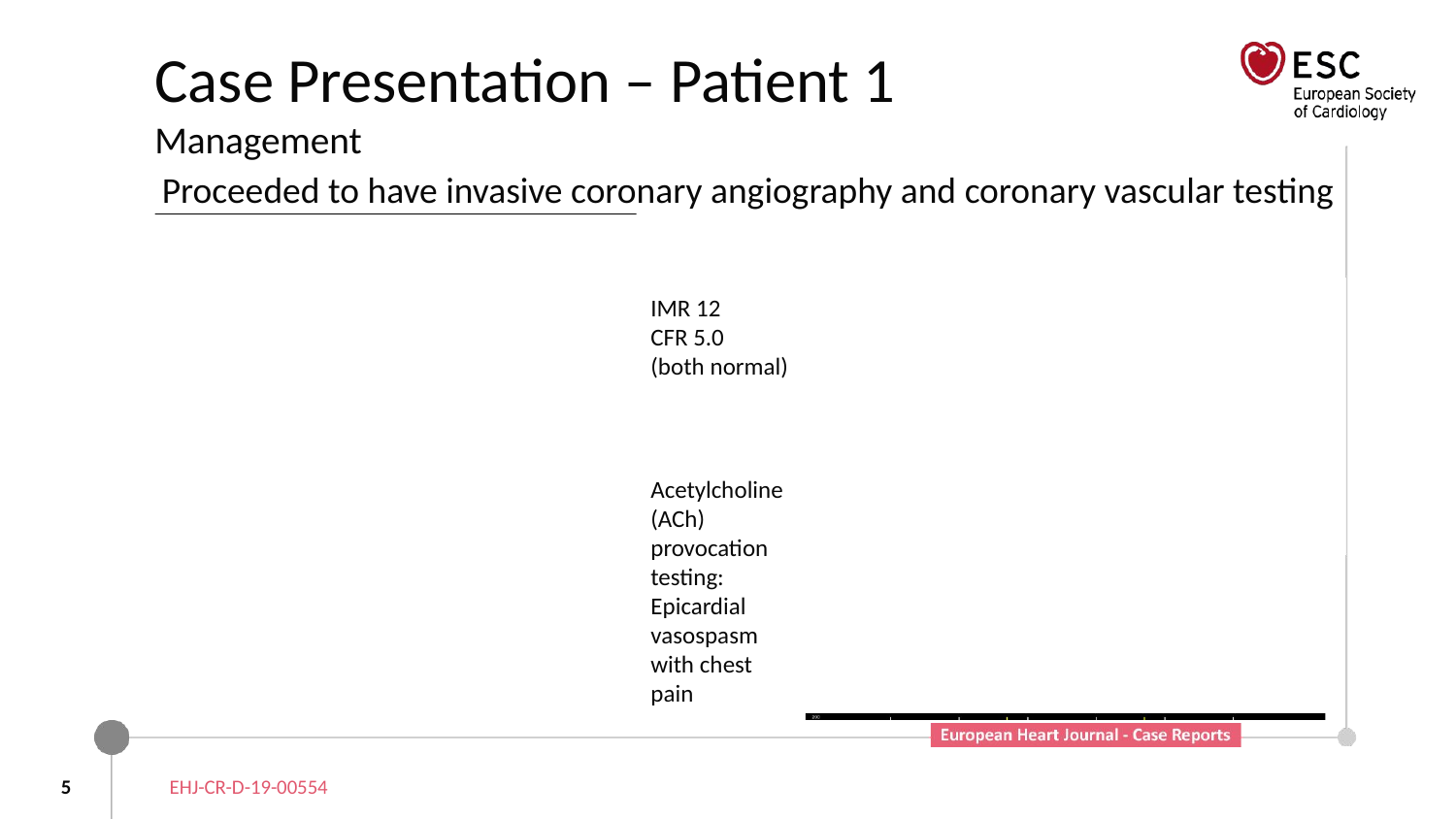

# Case Presentation – Patient 1 Management
Proceeded to have invasive coronary angiography and coronary vascular testing
IMR 12
CFR 5.0
(both normal)
Acetylcholine (ACh) provocation testing: Epicardial vasospasm with chest pain
5
EHJ-CR-D-19-00554

## Slide 6
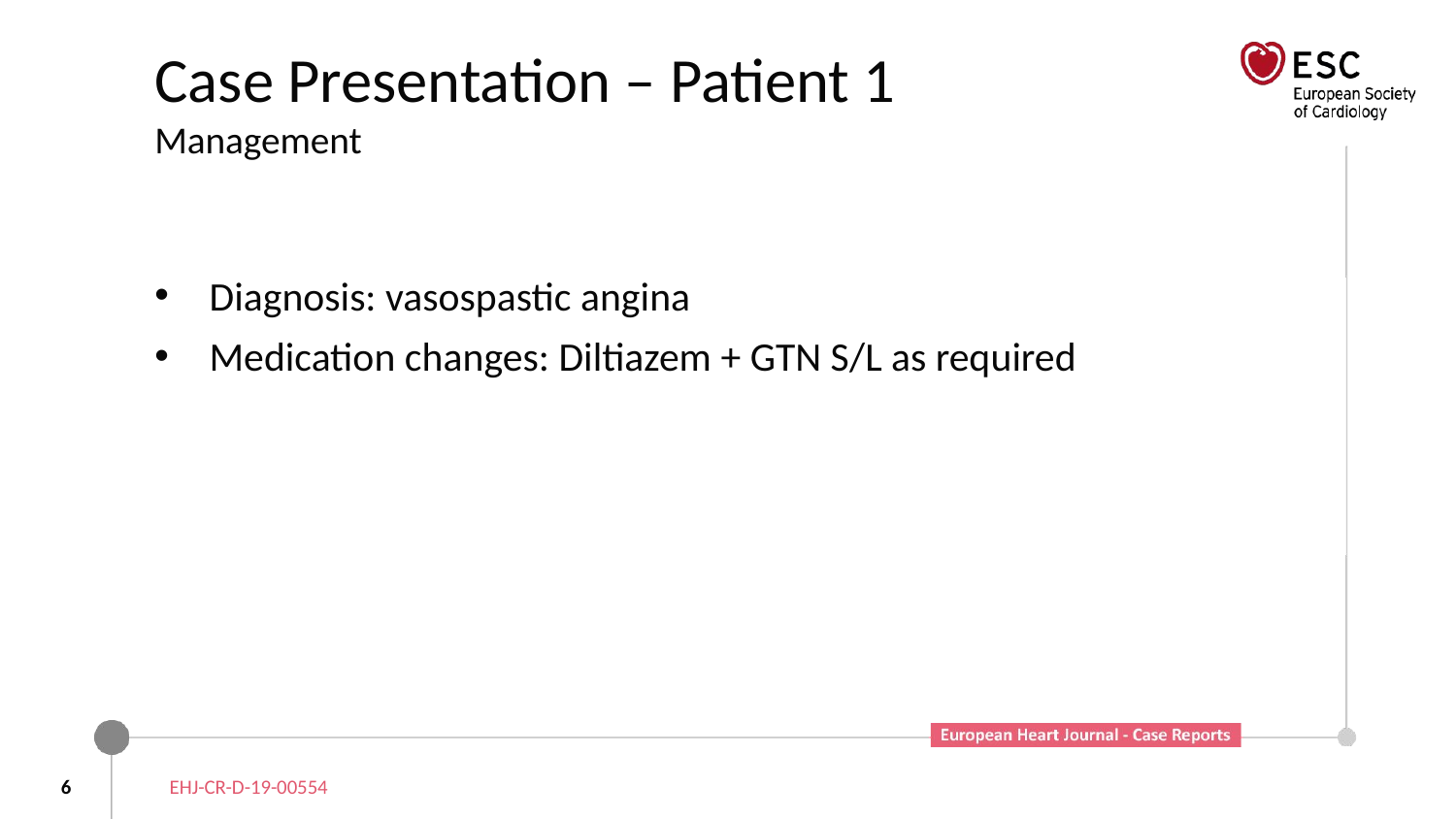

# Case Presentation – Patient 1 Management
Diagnosis: vasospastic angina
Medication changes: Diltiazem + GTN S/L as required
6
EHJ-CR-D-19-00554

## Slide 7
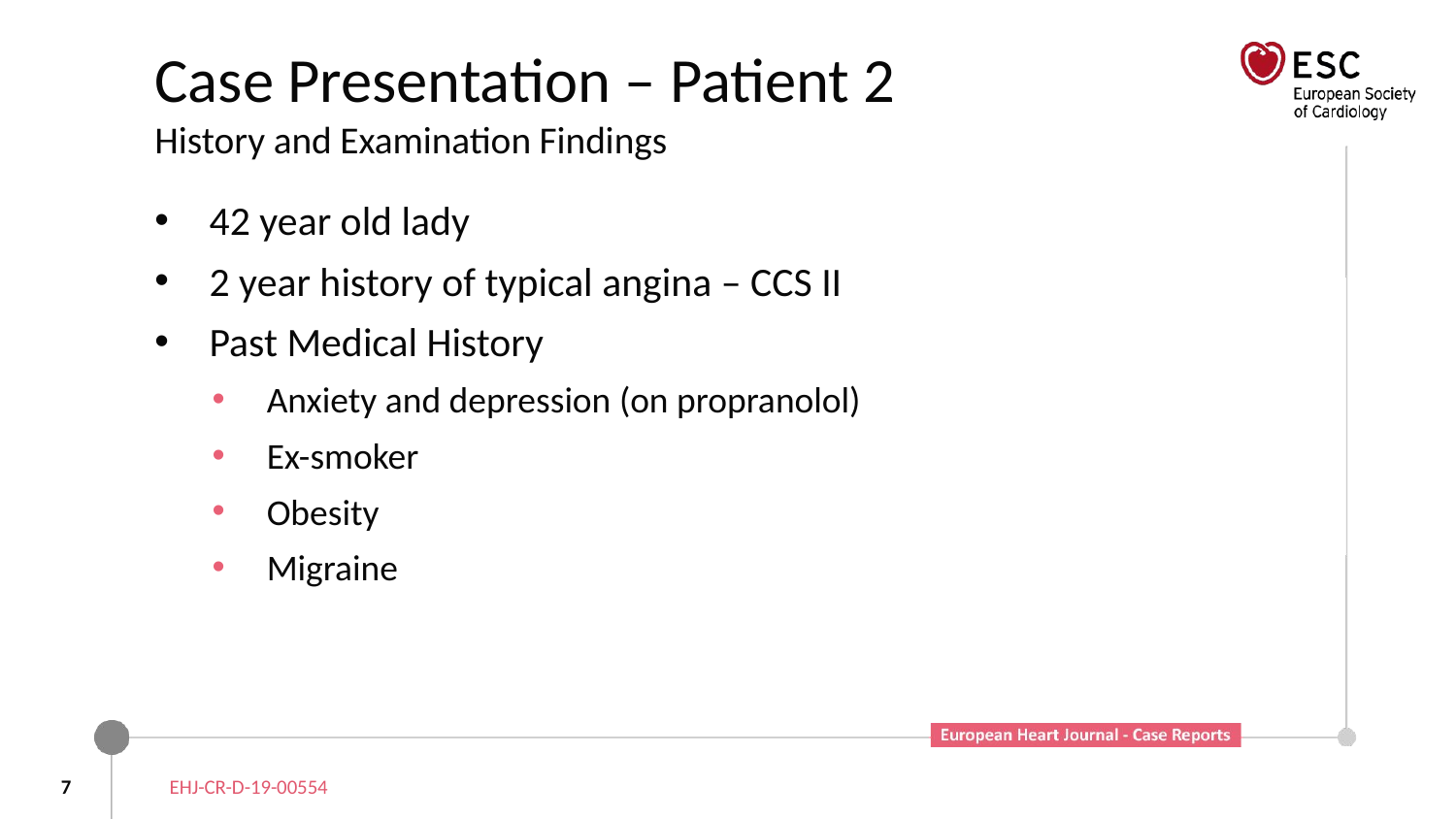

# Case Presentation – Patient 2 History and Examination Findings
42 year old lady
2 year history of typical angina – CCS II
Past Medical History
Anxiety and depression (on propranolol)
Ex-smoker
Obesity
Migraine
7
EHJ-CR-D-19-00554

## Slide 8
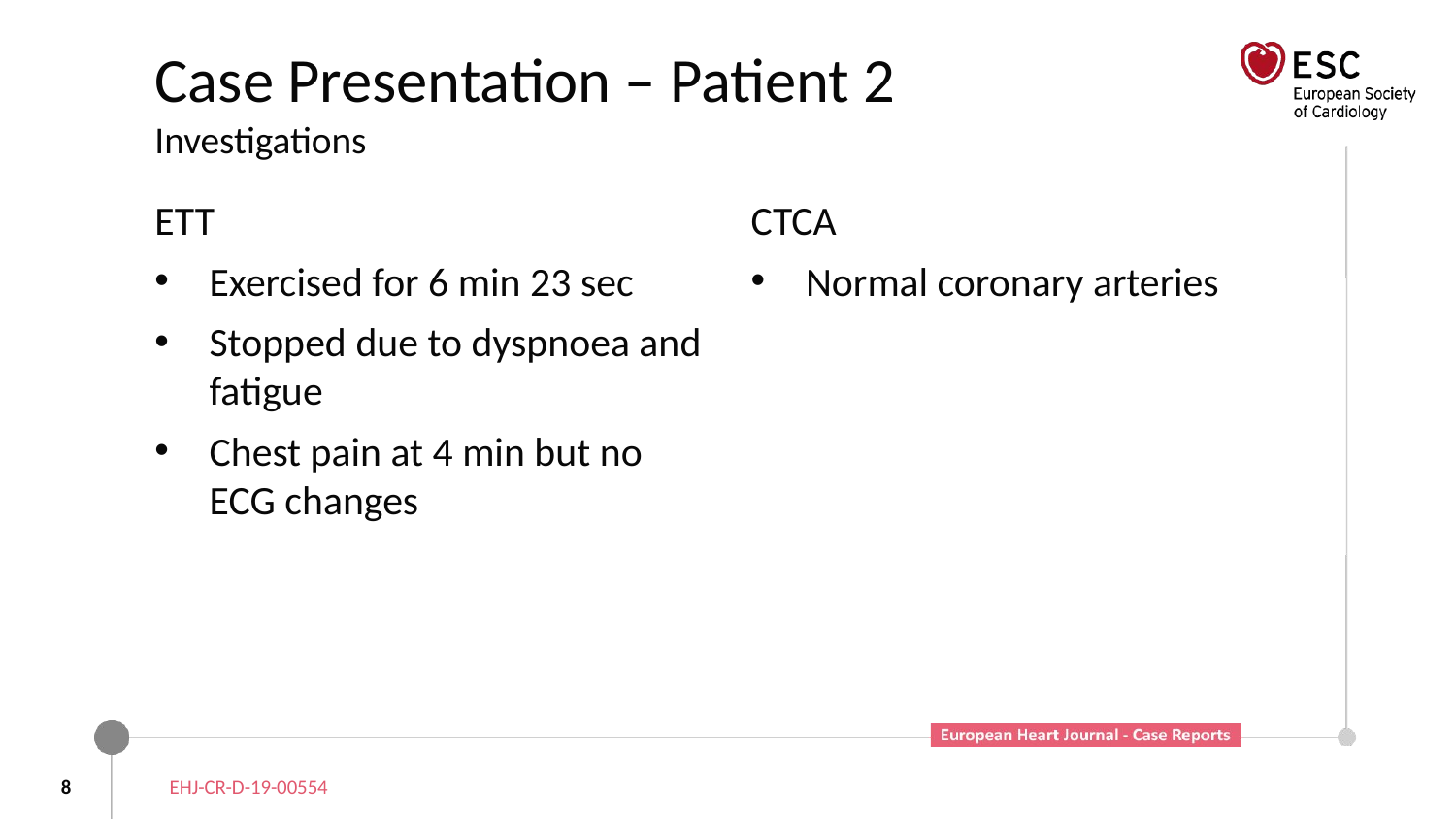

# Case Presentation – Patient 2 Investigations
ETT
Exercised for 6 min 23 sec
Stopped due to dyspnoea and fatigue
Chest pain at 4 min but no ECG changes
CTCA
Normal coronary arteries
8
EHJ-CR-D-19-00554

## Slide 9
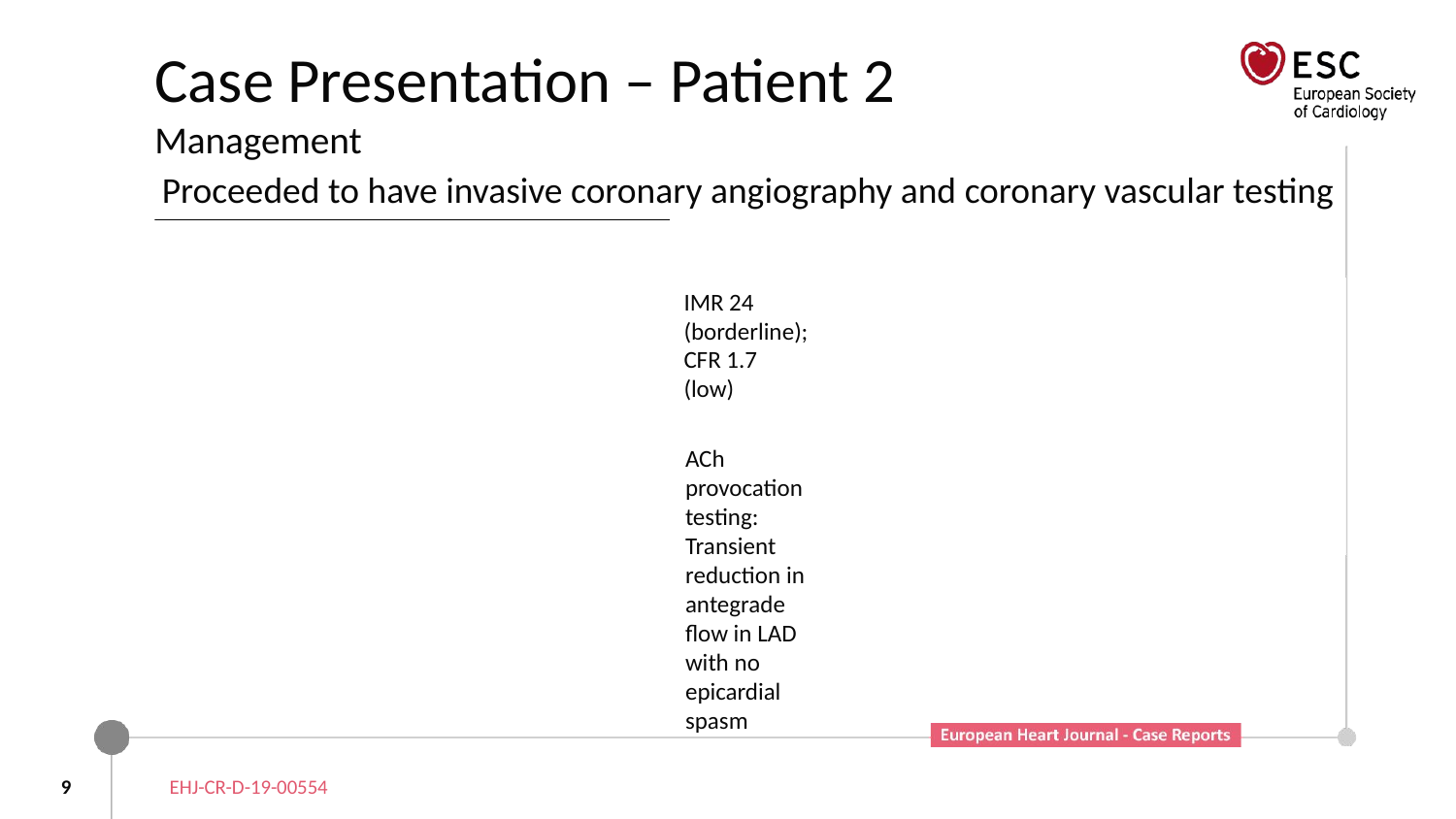

# Case Presentation – Patient 2 Management
Proceeded to have invasive coronary angiography and coronary vascular testing
IMR 24 (borderline); CFR 1.7 (low)
ACh provocation testing: Transient reduction in antegrade flow in LAD with no epicardial spasm
9
EHJ-CR-D-19-00554

## Slide 10
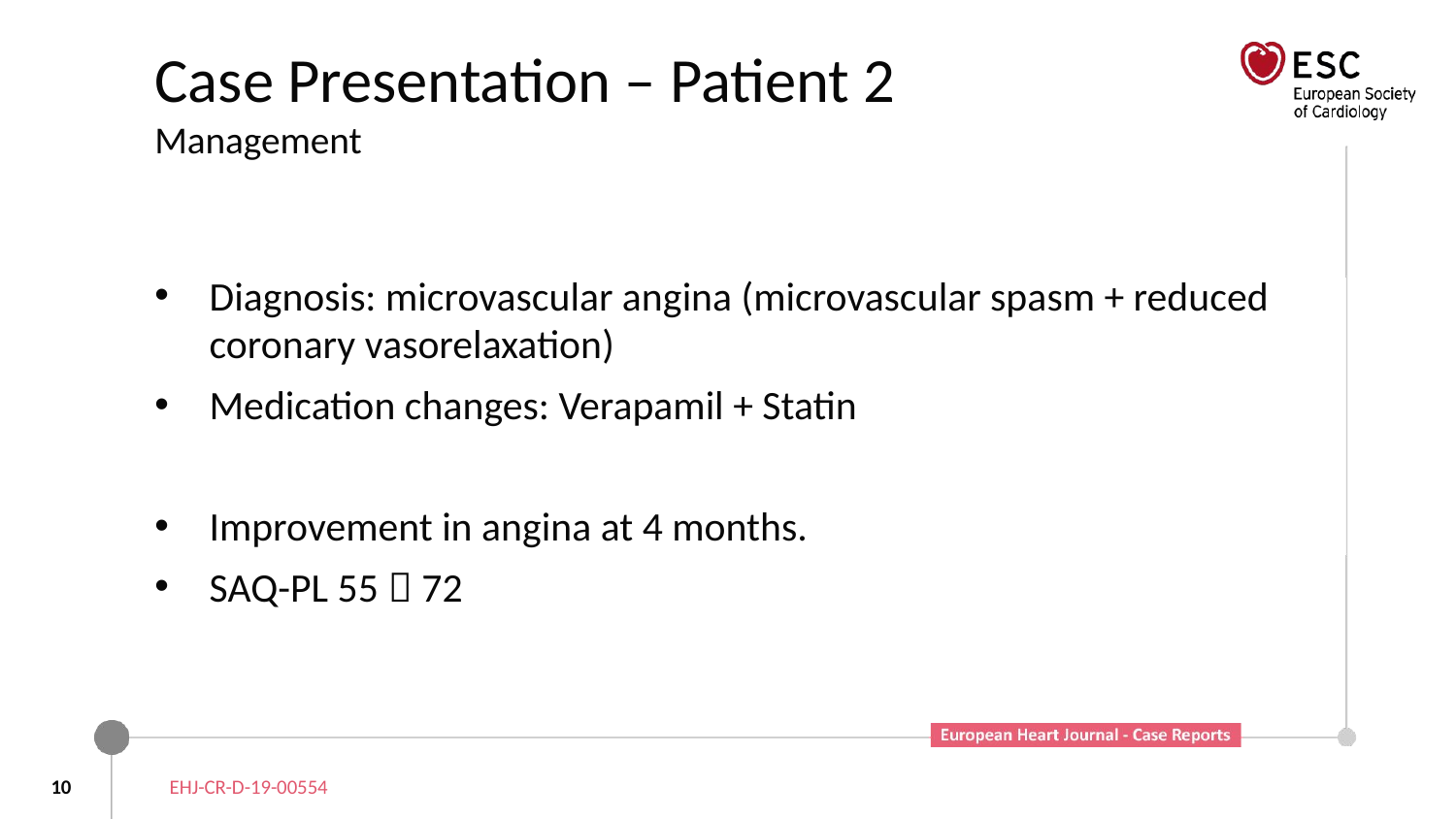

# Case Presentation – Patient 2 Management
Diagnosis: microvascular angina (microvascular spasm + reduced coronary vasorelaxation)
Medication changes: Verapamil + Statin
Improvement in angina at 4 months.
SAQ-PL 55  72
10
EHJ-CR-D-19-00554

## Slide 11
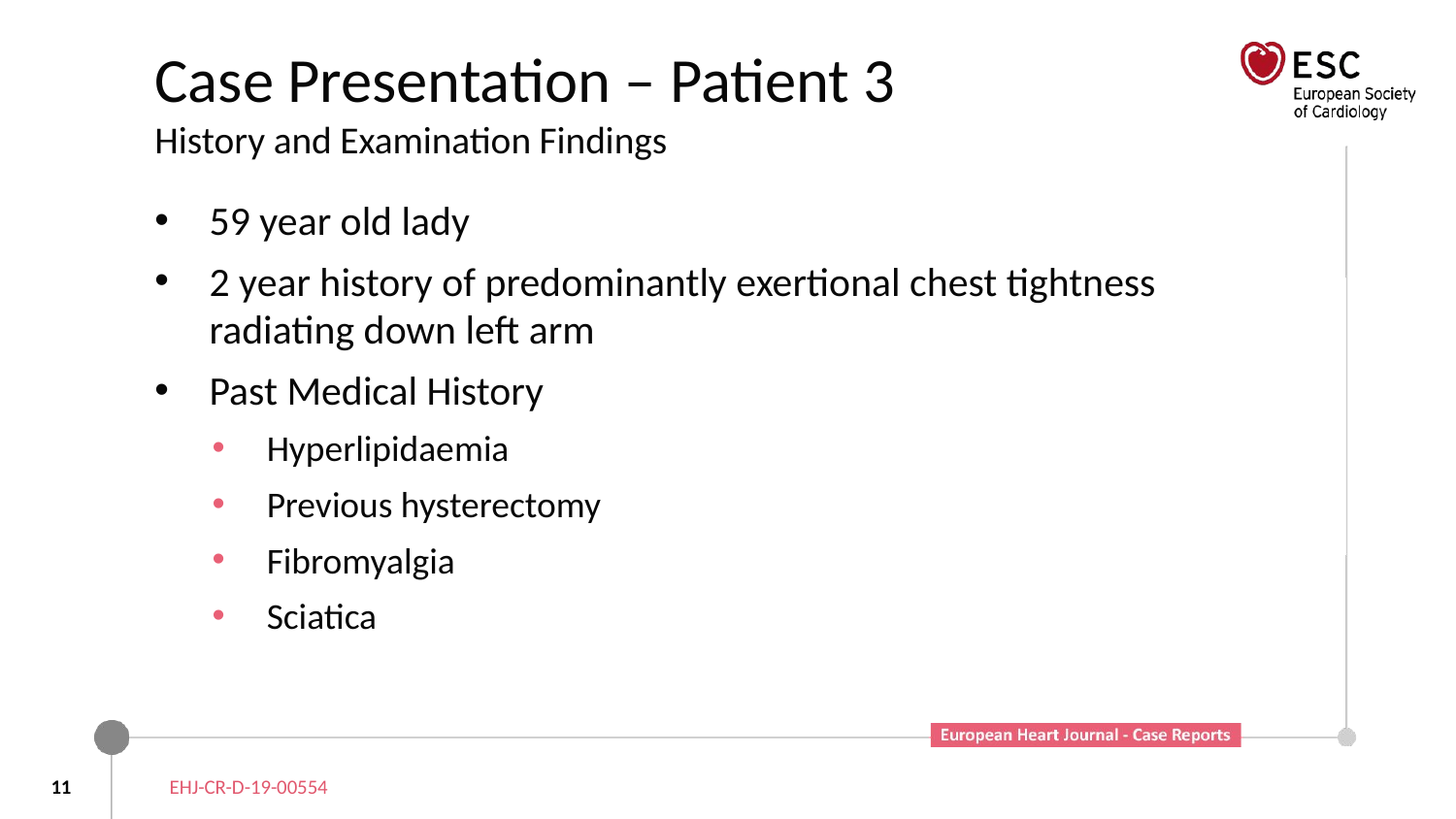

# Case Presentation – Patient 3 History and Examination Findings
59 year old lady
2 year history of predominantly exertional chest tightness radiating down left arm
Past Medical History
Hyperlipidaemia
Previous hysterectomy
Fibromyalgia
Sciatica
11
EHJ-CR-D-19-00554

## Slide 12
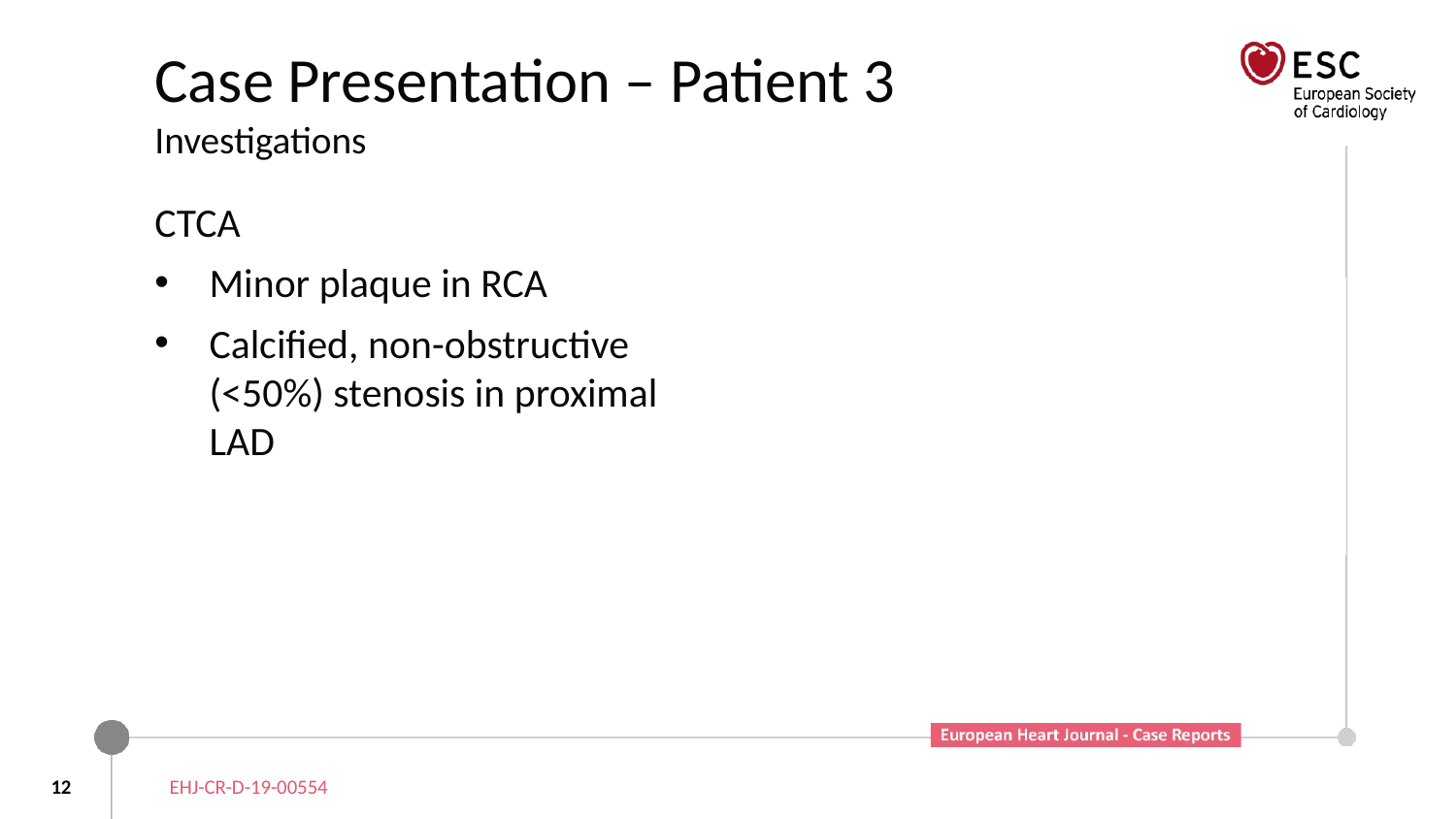

# Case Presentation – Patient 3 Investigations
CTCA
Minor plaque in RCA
Calcified, non-obstructive (<50%) stenosis in proximal LAD
12
EHJ-CR-D-19-00554

## Slide 13
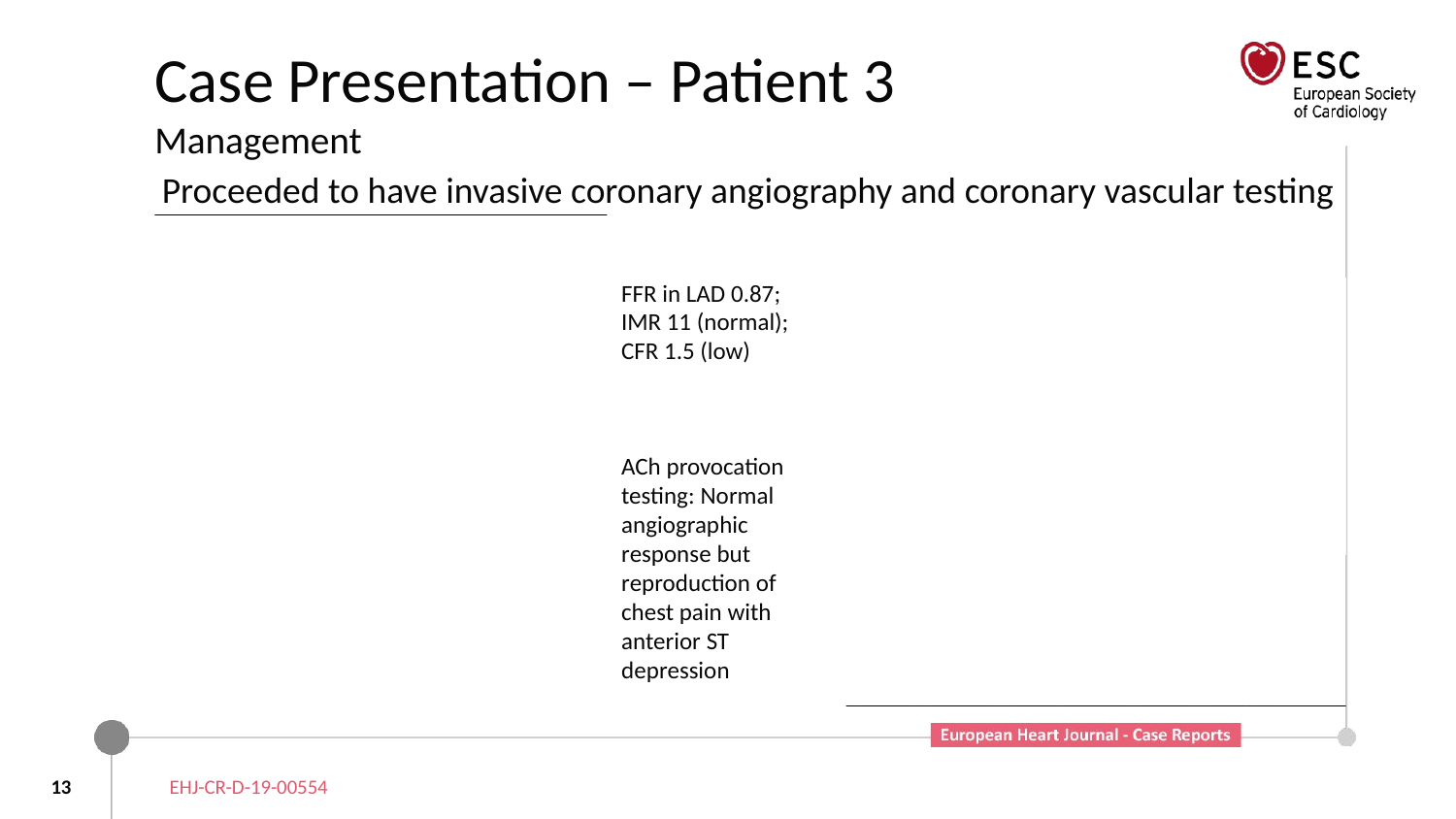

# Case Presentation – Patient 3 Management
Proceeded to have invasive coronary angiography and coronary vascular testing
FFR in LAD 0.87;
IMR 11 (normal);
CFR 1.5 (low)
ACh provocation testing: Normal angiographic response but reproduction of chest pain with anterior ST depression
13
EHJ-CR-D-19-00554

## Slide 14
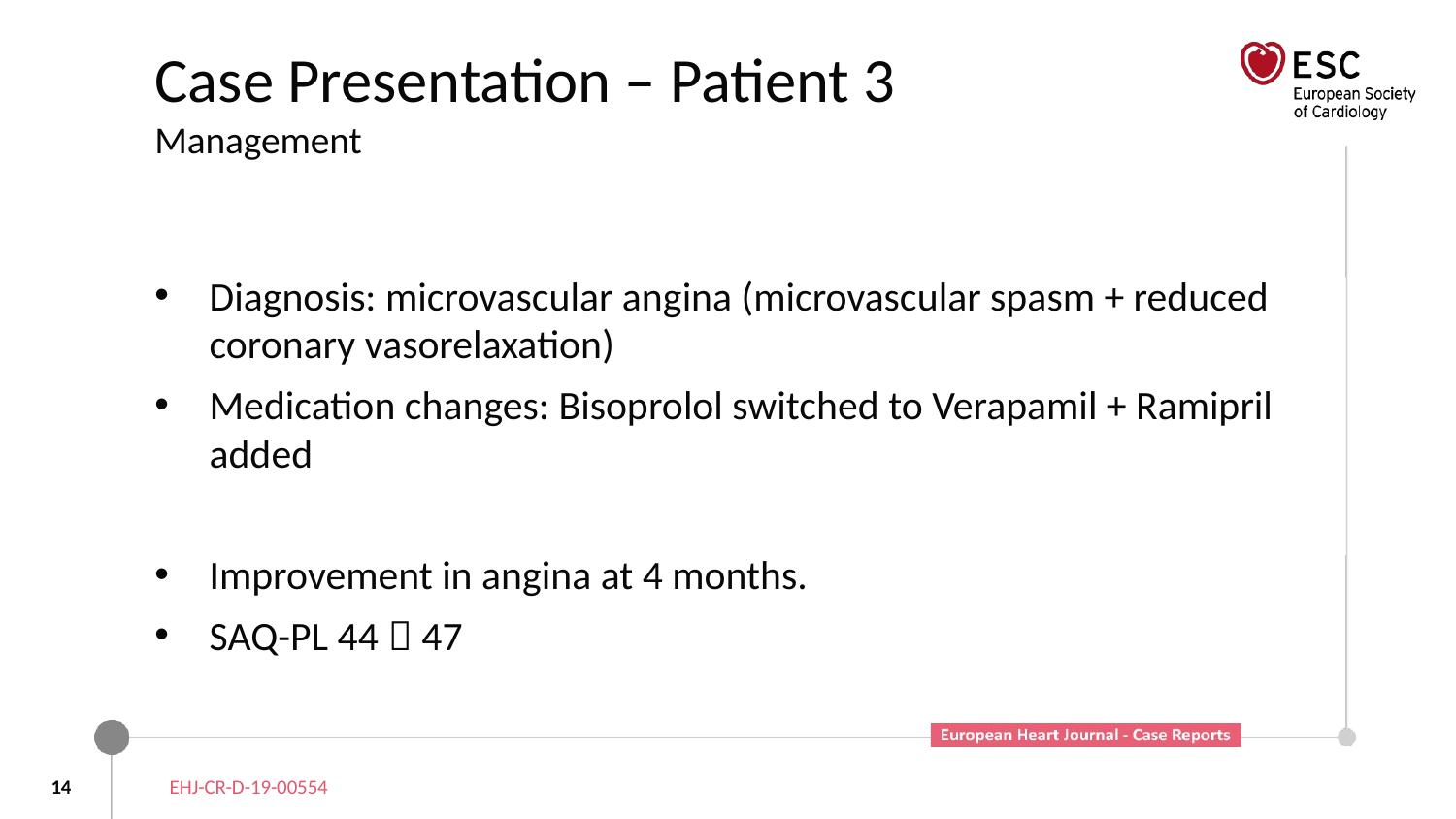

# Case Presentation – Patient 3 Management
Diagnosis: microvascular angina (microvascular spasm + reduced coronary vasorelaxation)
Medication changes: Bisoprolol switched to Verapamil + Ramipril added
Improvement in angina at 4 months.
SAQ-PL 44  47
14
EHJ-CR-D-19-00554

## Slide 15
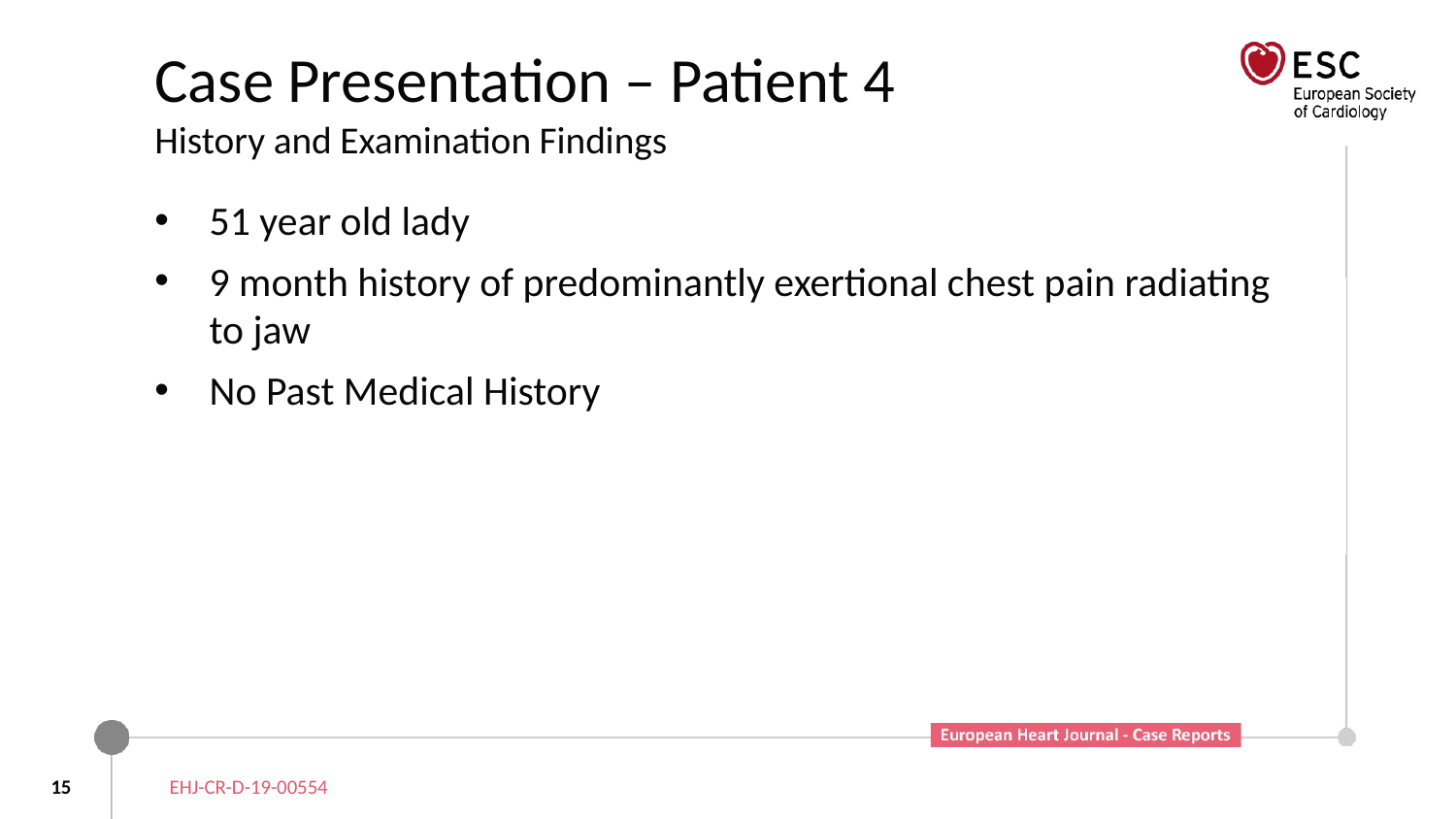

# Case Presentation – Patient 4 History and Examination Findings
51 year old lady
9 month history of predominantly exertional chest pain radiating to jaw
No Past Medical History
15
EHJ-CR-D-19-00554

## Slide 16
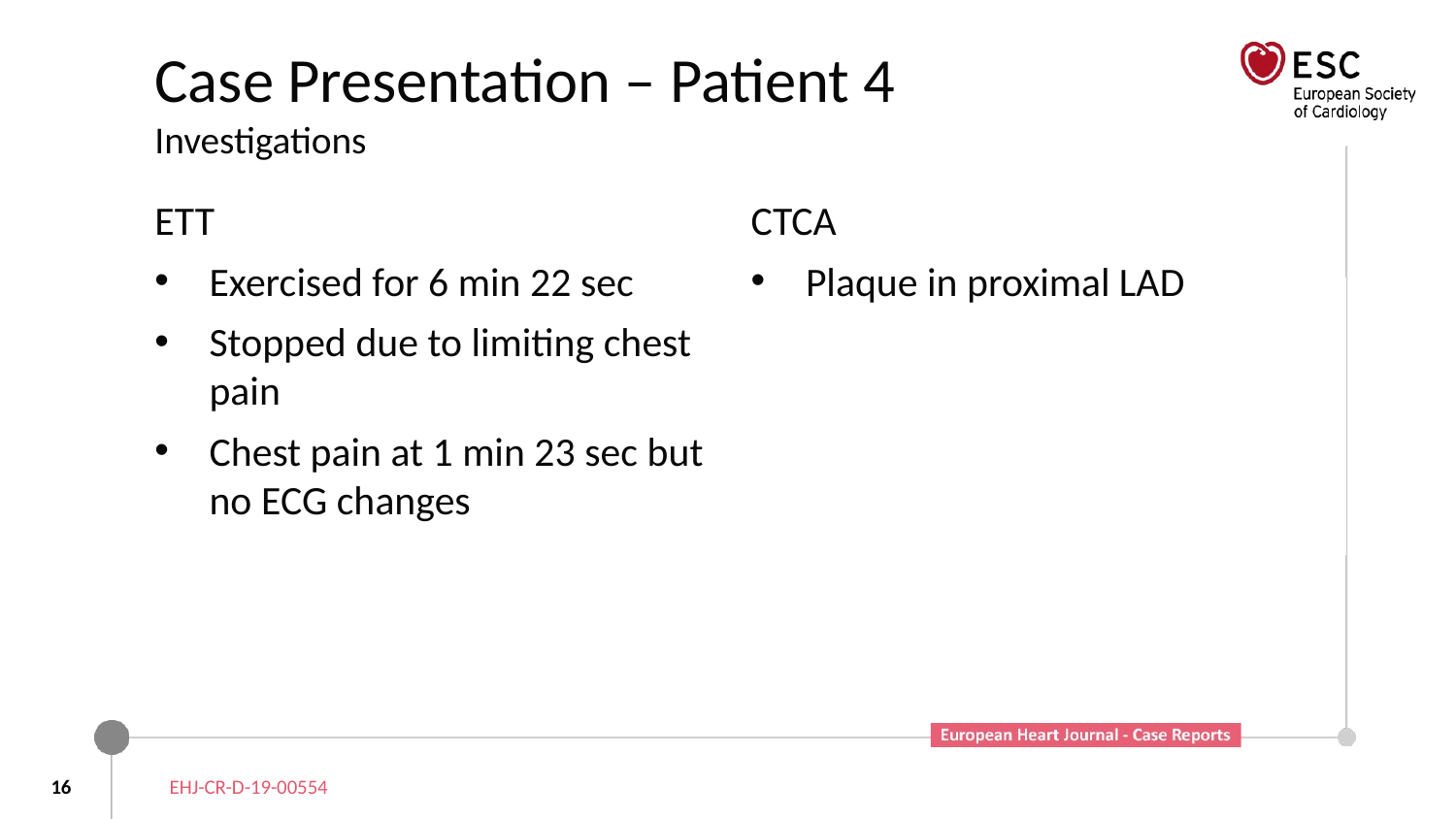

# Case Presentation – Patient 4 Investigations
ETT
Exercised for 6 min 22 sec
Stopped due to limiting chest pain
Chest pain at 1 min 23 sec but no ECG changes
CTCA
Plaque in proximal LAD
16
EHJ-CR-D-19-00554

## Slide 17
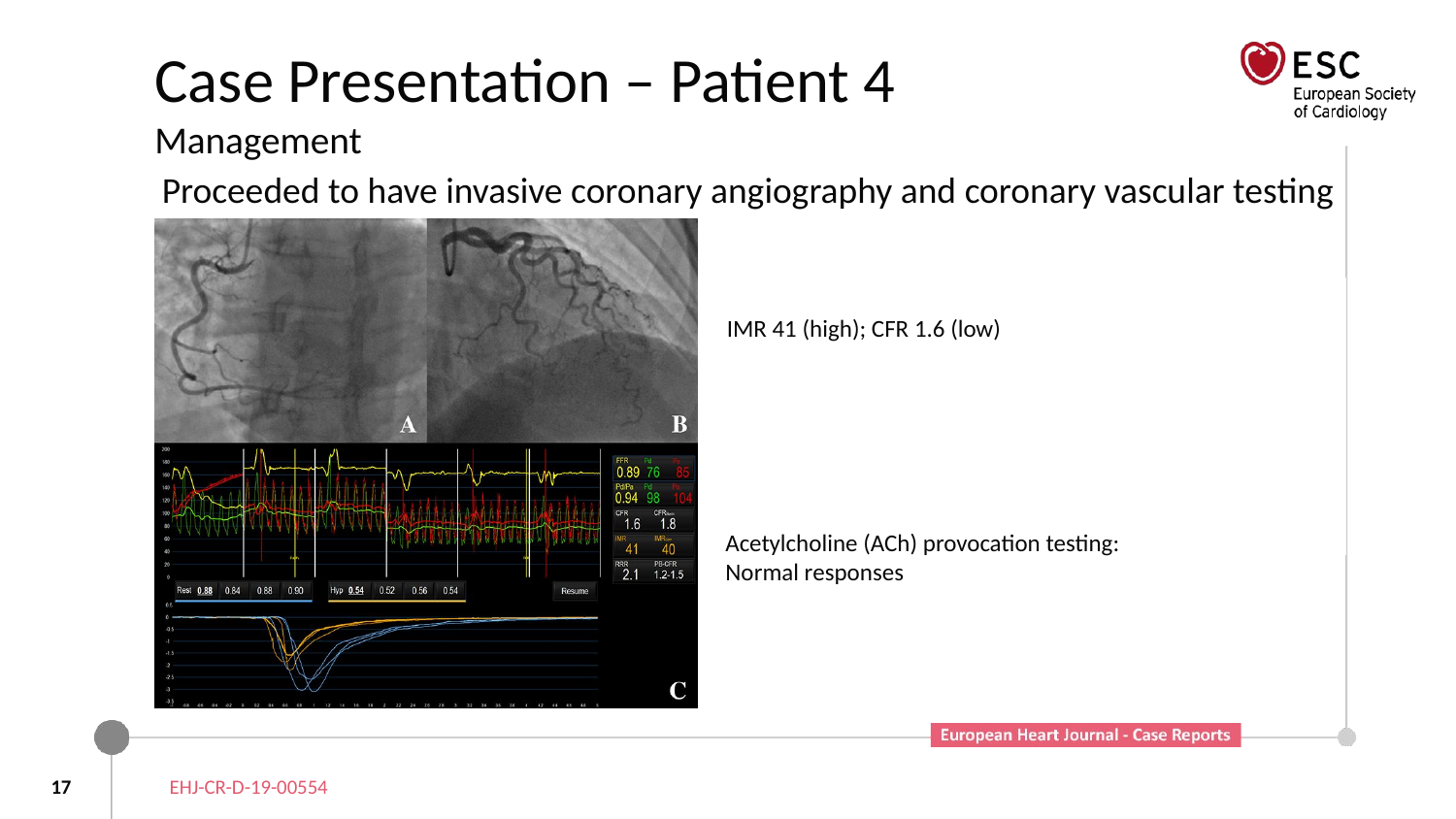

# Case Presentation – Patient 4 Management
Proceeded to have invasive coronary angiography and coronary vascular testing
IMR 41 (high); CFR 1.6 (low)
Acetylcholine (ACh) provocation testing: Normal responses
17
EHJ-CR-D-19-00554

## Slide 18
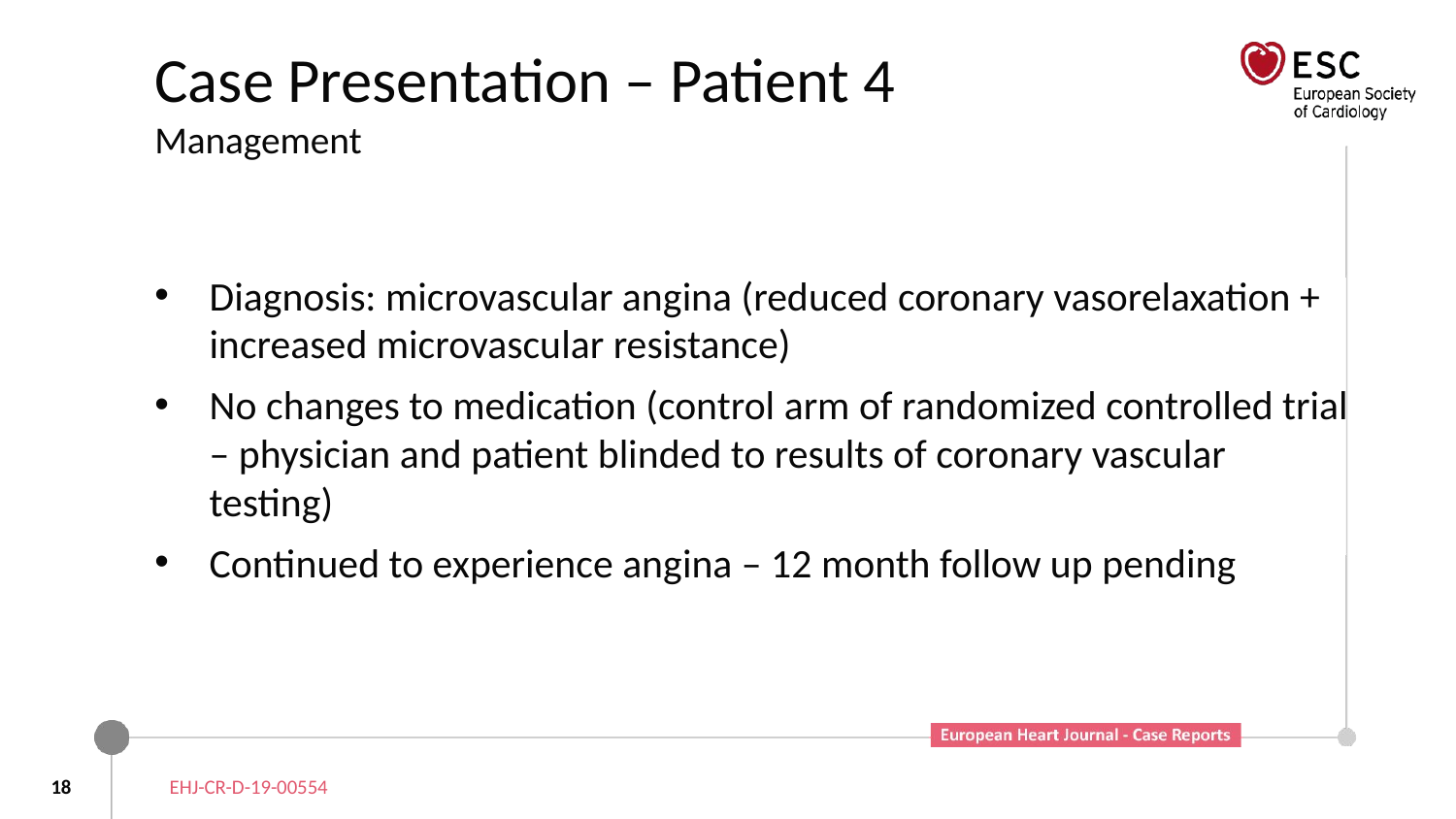

# Case Presentation – Patient 4 Management
Diagnosis: microvascular angina (reduced coronary vasorelaxation + increased microvascular resistance)
No changes to medication (control arm of randomized controlled trial – physician and patient blinded to results of coronary vascular testing)
Continued to experience angina – 12 month follow up pending
18
EHJ-CR-D-19-00554

## Slide 19
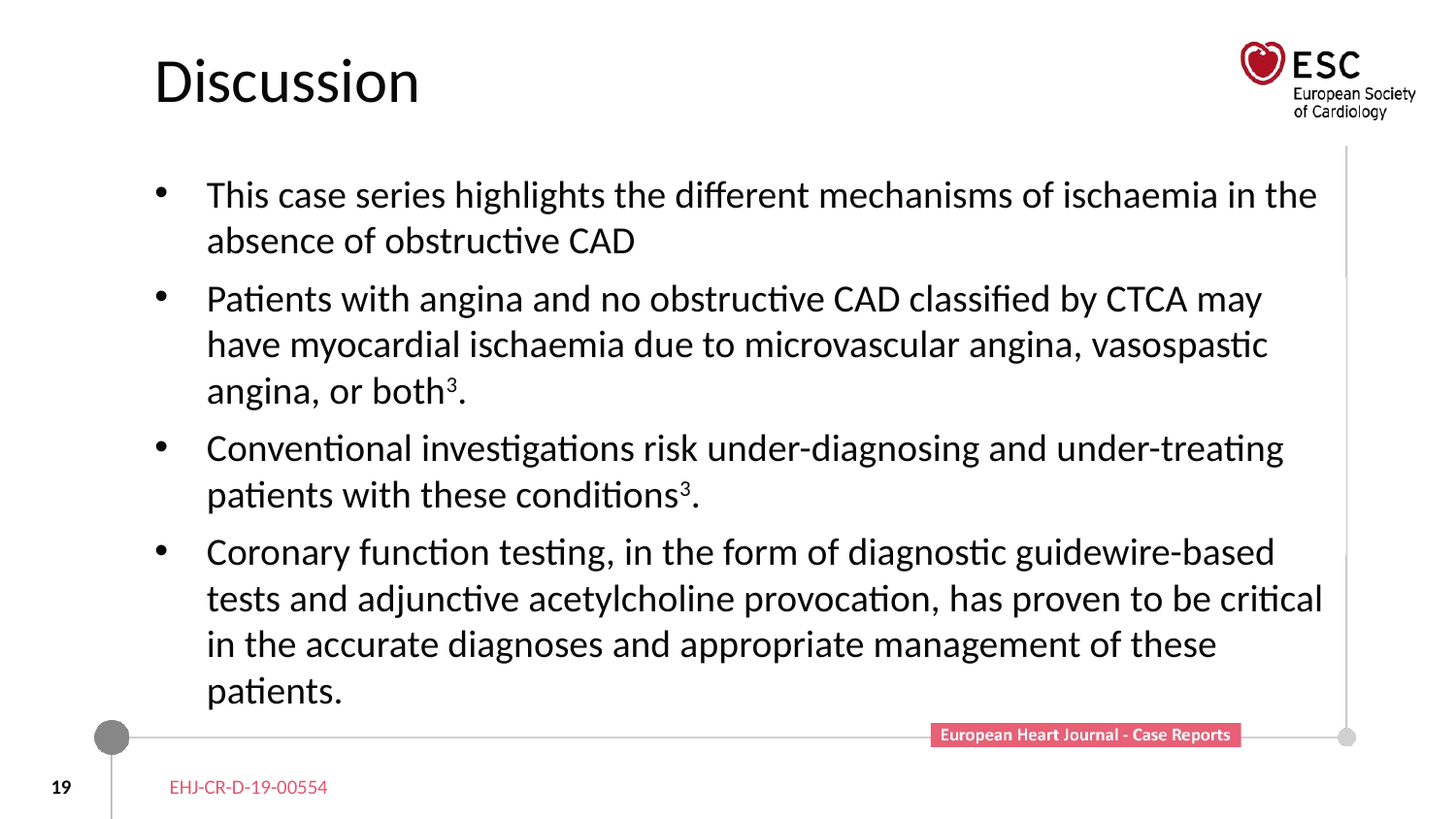

# Discussion
This case series highlights the different mechanisms of ischaemia in the absence of obstructive CAD
Patients with angina and no obstructive CAD classified by CTCA may have myocardial ischaemia due to microvascular angina, vasospastic angina, or both3.
Conventional investigations risk under-diagnosing and under-treating patients with these conditions3.
Coronary function testing, in the form of diagnostic guidewire-based tests and adjunctive acetylcholine provocation, has proven to be critical in the accurate diagnoses and appropriate management of these patients.
19
EHJ-CR-D-19-00554

## Slide 20
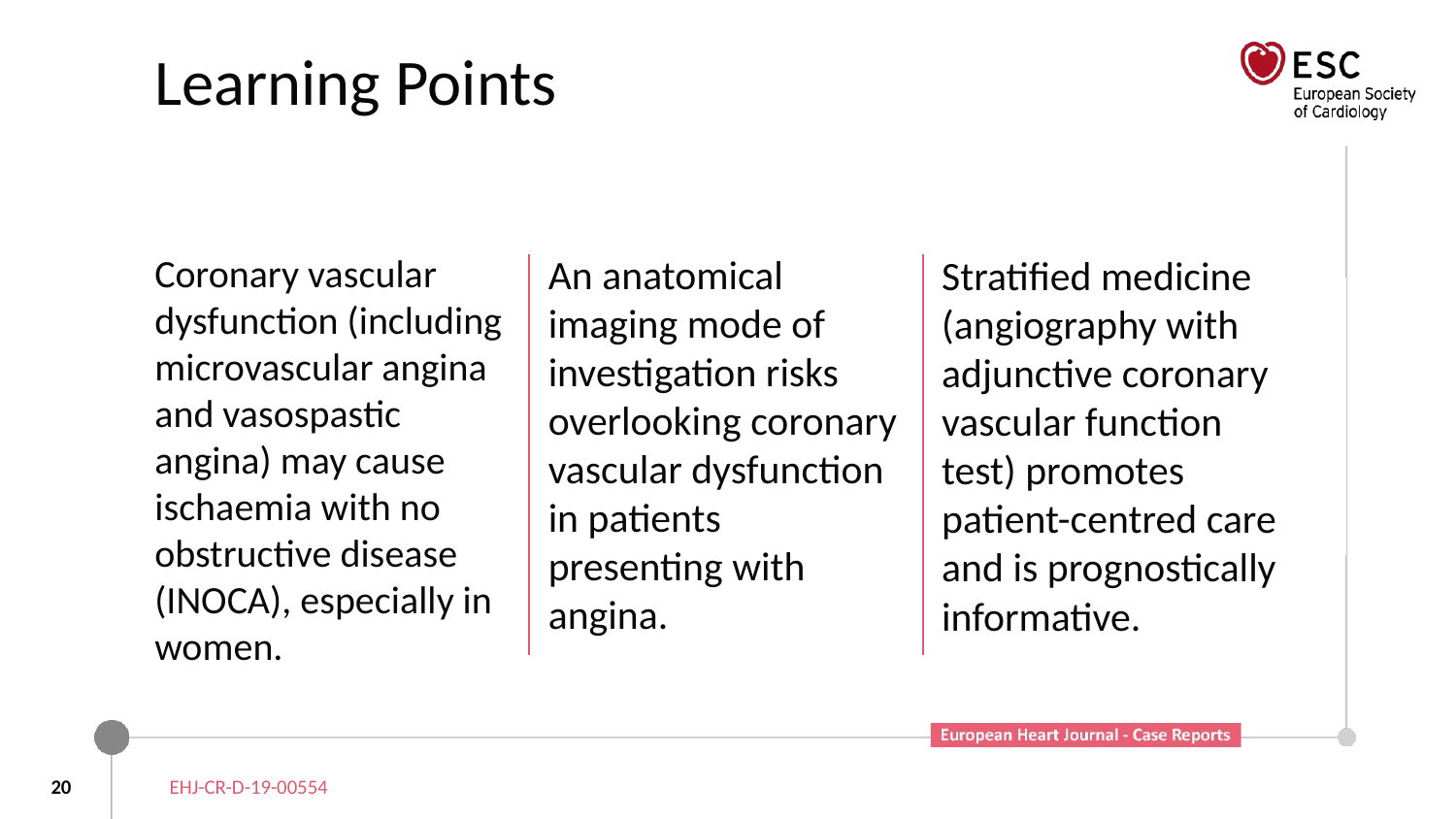

# Learning Points
Coronary vascular dysfunction (including microvascular angina and vasospastic angina) may cause ischaemia with no obstructive disease (INOCA), especially in women.
An anatomical imaging mode of investigation risks overlooking coronary vascular dysfunction in patients presenting with angina.
Stratified medicine (angiography with adjunctive coronary vascular function test) promotes patient-centred care and is prognostically informative.
20
EHJ-CR-D-19-00554

## Slide 21
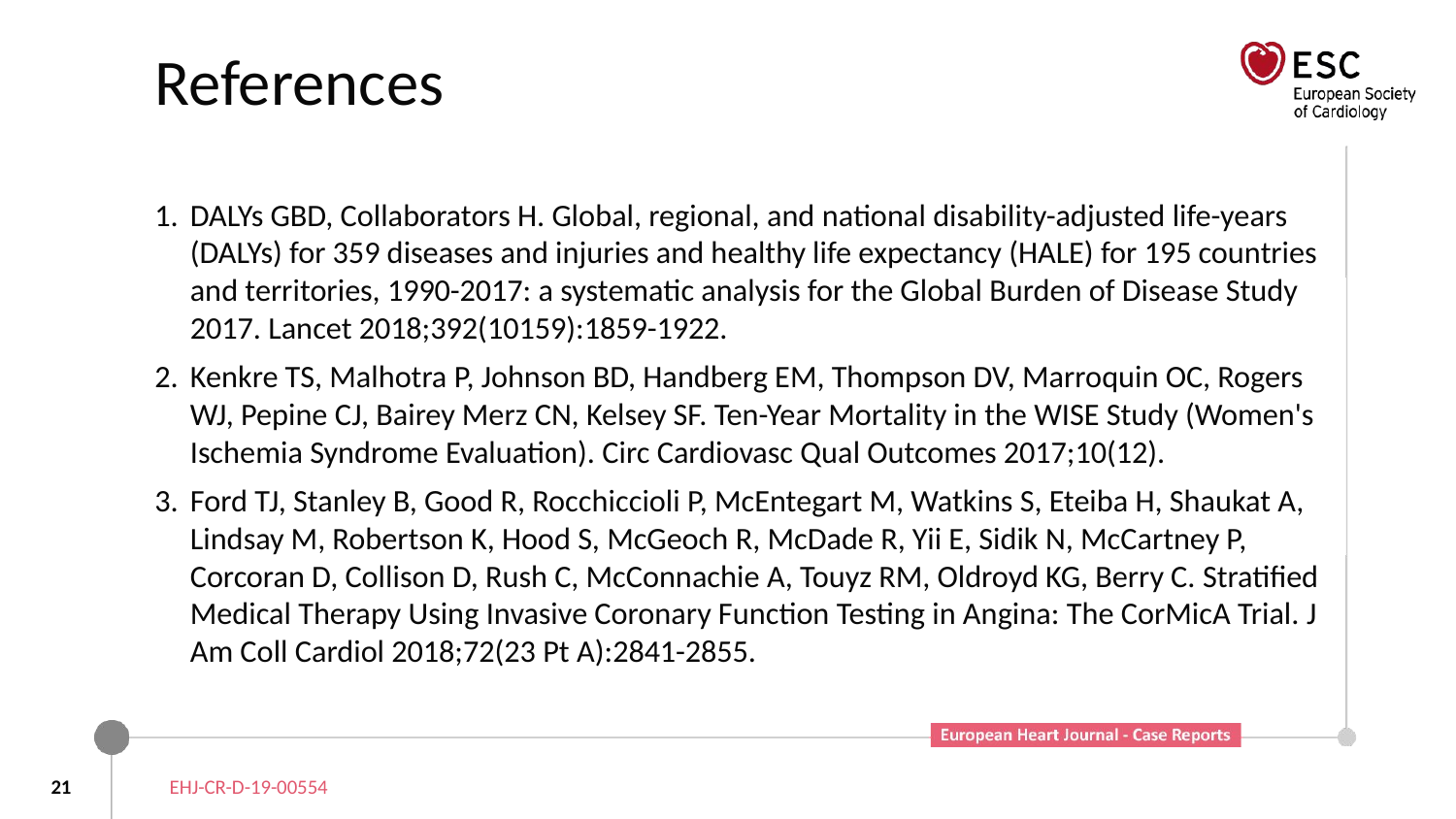

# References
DALYs GBD, Collaborators H. Global, regional, and national disability-adjusted life-years (DALYs) for 359 diseases and injuries and healthy life expectancy (HALE) for 195 countries and territories, 1990-2017: a systematic analysis for the Global Burden of Disease Study 2017. Lancet 2018;392(10159):1859-1922.
Kenkre TS, Malhotra P, Johnson BD, Handberg EM, Thompson DV, Marroquin OC, Rogers WJ, Pepine CJ, Bairey Merz CN, Kelsey SF. Ten-Year Mortality in the WISE Study (Women's Ischemia Syndrome Evaluation). Circ Cardiovasc Qual Outcomes 2017;10(12).
Ford TJ, Stanley B, Good R, Rocchiccioli P, McEntegart M, Watkins S, Eteiba H, Shaukat A, Lindsay M, Robertson K, Hood S, McGeoch R, McDade R, Yii E, Sidik N, McCartney P, Corcoran D, Collison D, Rush C, McConnachie A, Touyz RM, Oldroyd KG, Berry C. Stratified Medical Therapy Using Invasive Coronary Function Testing in Angina: The CorMicA Trial. J Am Coll Cardiol 2018;72(23 Pt A):2841-2855.
21
EHJ-CR-D-19-00554
